# Urea/acrylamide-assisted nitrogen-functionalized rice husk biochar for Cd(ii) removal: adsorption performance and interaction insights

**DOI:** 10.1039/d6ra08271h

**Published:** 2026-10-08

**Authors:** Adel A. El-Zahhar, Majed M. Alghamdi

**Affiliations:** a Department of Chemistry, Faculty of Science, King Khalid University P. O. Box 9004 Abha 61413 Saudi Arabia Azmasnew26@hotmail.com

## Abstract

Cadmium-bearing wastewater remains a major environmental concern, requiring effective sorbents that can operate under complex aqueous conditions. Herein, urea/acrylamide-assisted nitrogen-functionalized activated rice husk biochar, denoted UA-NARB, was synthesized through preliminary carbonization followed by K_2_CO_3_-assisted activation and nitrogen functionalization using urea and acrylamide as nitrogen-containing precursors. XRD, FTIR, SEM, N_2_ adsorption–desorption, DLS, and zeta potential analyses revealed a heterogeneous carbon–mineral sorbent with disordered carbon domains, O-/N-containing functionalities, accessible mesoporosity, and a moderately negative surface charge. Cd(ii) adsorption was strongly pH-dependent, reaching 94.5% removal at pH 6, which was selected as the working condition. A sorbent dose of 2.0 g L^−1^ provided an effective balance between Cd(ii) removal and material utilization. Kinetic analysis showed rapid initial uptake followed by gradual equilibration, with the pseudo-second-order model providing the best empirical description. Equilibrium adsorption exhibited saturation-type behavior and was well described by the Langmuir model, with a maximum adsorption capacity of 14.6 mg g^−1^. Thermodynamic parameters indicated spontaneous and endothermic Cd(ii) uptake. From an application perspective, UA-NARB showed efficient regenerability, with 1.0 M H_2_SO_4_ desorbing 92.7% of adsorbed Cd(ii), and maintained 80.7% Cd(ii) removal from synthetic multimetal wastewater. The adsorption process is proposed to proceed through cooperative contributions from pH-regulated electrostatic attraction, interactions with polar surface functionalities and mineral-associated sites, and diffusion through accessible mesoporous domains. These results position UA-NARB as a rice-husk-derived sorbent with practical potential for Cd(ii)-contaminated wastewater treatment.

## Abstract 2

Cadmium-bearing wastewater remains a major environmental concern, requiring effective sorbents that can operate under complex aqueous conditions.

## Graphical abstract

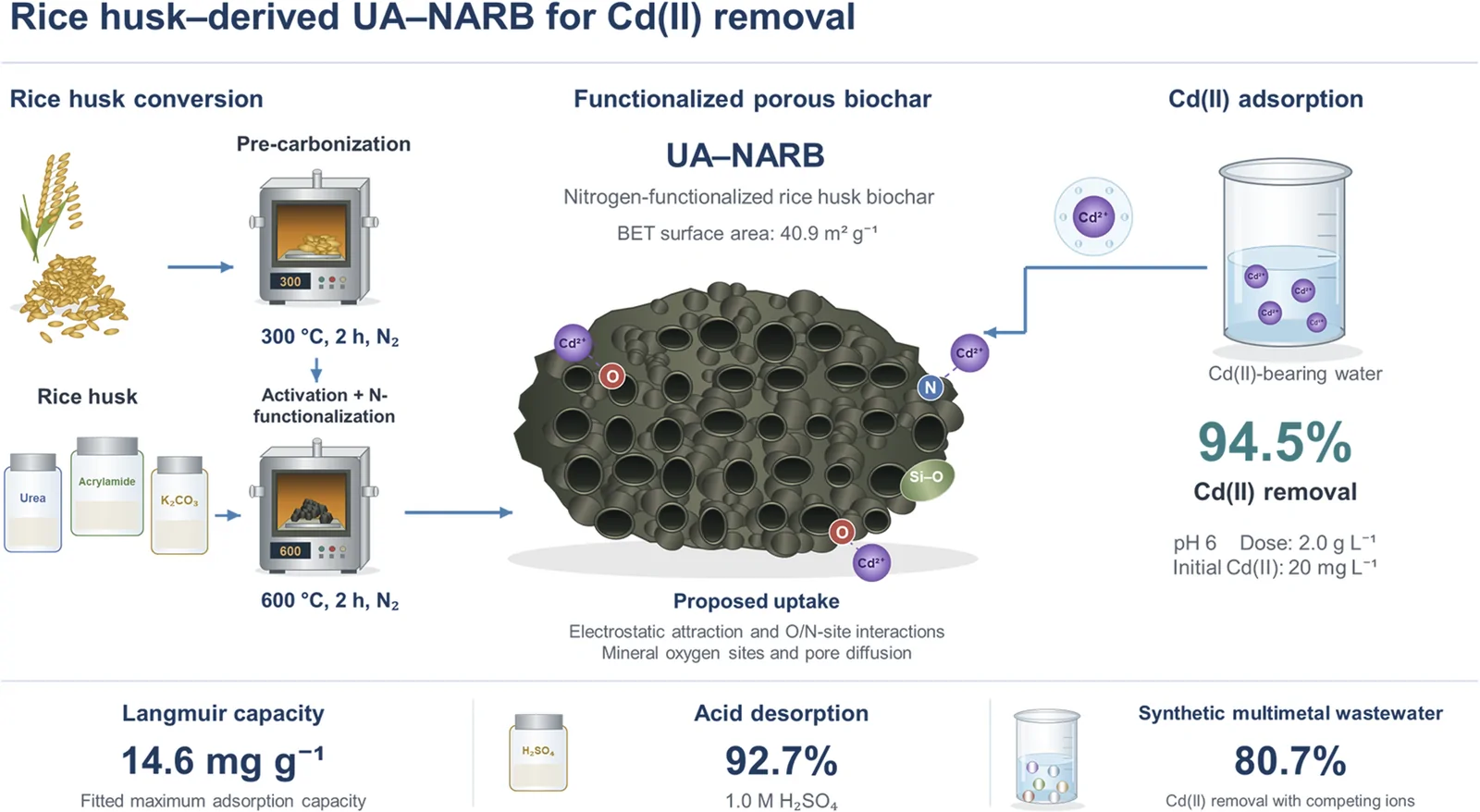

## 1. Introduction

Cadmium is among the most hazardous heavy-metal pollutants encountered in industrial effluents and contaminated water systems because of its high toxicity, environmental persistence, and non-biodegradable nature.^1,2^ Unlike many organic contaminants that can be degraded or transformed under environmental conditions, Cd(ii) remains chemically persistent and can migrate through water, soil, and biological pathways, thereby posing risks to ecosystems and human health even at low concentrations.^3,4^ Major anthropogenic sources of Cd(ii) contamination include electroplating, mining, battery manufacturing, pigment production, phosphate fertilizer industries, metal finishing, and improper discharge of industrial wastewater.^5–7^ Cadmium exposure is associated with severe health risks, including renal dysfunction, skeletal damage, and carcinogenic effects, and its concentration in drinking water is therefore strictly regulated, with the World Health Organization recommending a maximum guideline value of 0.003 mg L^−1^ for Cd in drinking water.^8,9^ Therefore, the development of efficient, low-cost, and environmentally compatible treatment technologies for Cd(ii)-containing water remains an important research priority in environmental chemistry and wastewater engineering.

Several treatment technologies have been explored for removing Cd(ii) and other heavy metals from aqueous systems, including chemical precipitation, membrane filtration, ion exchange, electrochemical treatment, coagulation–flocculation, and adsorption.^10–14^ Although many of these methods can achieve effective metal removal under controlled conditions, their wider application may be limited by high operational cost, secondary sludge generation, membrane fouling, chemical consumption, difficult regeneration, or reduced efficiency at low metal concentrations.^15–17^ In this context, adsorption has attracted considerable attention because of its operational simplicity, design flexibility, relatively low energy demand, and ability to treat dilute metal-bearing solutions.^3–5^ The effectiveness of adsorption, however, depends strongly on the physicochemical properties of the applied sorbent, including surface area, pore architecture, surface charge, functional groups, mineral composition, and chemical stability.^4–7^ Consequently, the design of rice-husk-derived sorbent with accessible binding sites and favorable surface chemistry is central to improving Cd(ii) removal from water.

Biochar-based sorbents derived from agricultural residues have emerged as attractive alternatives to conventional adsorbents because they combine waste valorization, carbon-rich structure, tunable surface chemistry, and potential environmental sustainability.^3,4,18,19^ Among agricultural residues, rice husk is particularly promising because it is produced in large quantities as an agricultural by-product of rice milling, and its conversion into functional biochar-based sorbents provides a sustainable route for coupling agricultural waste valorization with wastewater remediation.^18–20^ Its intrinsic composition, which combines lignocellulosic organic matter with a silica-rich inorganic fraction, is advantageous for producing carbon–mineral sorbents containing oxygenated groups, aromatic carbon domains, mineral oxygen sites, and porous structures relevant to Cd(ii) uptake.^5,20,21^ However, pristine biochar often shows limited adsorption efficiency because of insufficient surface area, low density of active sites, weak surface polarity, or blocked pore channels.^4,16,18^ Therefore, chemical activation and heteroatom functionalization have been widely adopted to improve the accessibility, surface reactivity, and binding affinity of biochar-derived sorbents.^4,18,22^

Nitrogen functionalization is particularly useful for enhancing the adsorption potential of carbonaceous sorbents because nitrogen-containing groups can modify surface basicity, electron-donor capacity, surface polarity, and metal-binding affinity.^4,16,18^ Depending on the precursor and thermal treatment, different nitrogen-containing surface environments can be generated, which may influence metal uptake by modifying the local electronic and acid–base properties of the carbon surface.^4,18,19^ Urea and acrylamide are suitable nitrogen precursors because their thermal decomposition can release nitrogen-containing intermediates that promote the incorporation of N functionalities into the carbon matrix, thereby modifying the surface polarity and electron-donor character of the sorbent.^18,19,23,24^ In parallel, carbonate and alkali activators such as K_2_CO_3_ and KOH can promote pore development through chemical etching, gas evolution, and carbon-framework reconstruction, thereby improving adsorbate accessibility and diffusion pathways.^22–25^ During high-temperature activation, K_2_CO_3_ can contribute to pore development through carbon-framework etching, gas evolution, and reactions that widen blocked channels and generate more accessible micro/mesoporous structures.^23,24^ Accordingly, K_2_CO_3_-assisted activation and nitrogen functionalization can be integrated to provide accessible porosity and surface functionalities favorable for Cd(ii) adsorption.

Consequently, nitrogen functionalization combined with carbonate-assisted activation provides a rational route for preparing rice-husk-derived biochar with accessible pore structure and adsorption-relevant surface functionality. However, evaluating such materials requires more than reporting adsorption capacity; it also requires connecting synthesis-induced structural and chemical features with adsorption behavior under controlled and realistic aqueous conditions. Although many biochar-based Cd(ii) adsorbents have been reported, their discussion often remains capacity-centered, while fewer studies systematically connect precursor chemistry, activation-induced porosity, nitrogen/oxygen surface functionality, adsorption modeling, regeneration behavior, and real-wastewater performance within one structure–property–performance framework.^3–6^ Such an integrated approach is particularly important for agricultural-waste-derived sorbents, where moderate surface area may still produce effective adsorption if surface chemistry, mesoporosity, and ion-binding sites are favorably developed.^5,6^

Accordingly, the present study investigates the preparation, characterization, and Cd(ii) adsorption behavior of a urea/acrylamide-assisted nitrogen-functionalized activated rice husk biochar, designated UA-NARB. The sorbent was synthesized through preliminary rice husk carbonization followed by K_2_CO_3_-assisted activation and nitrogen functionalization using urea and acrylamide as nitrogen-containing precursors. The prepared material was characterized using XRD, FTIR, SEM, N_2_ adsorption–desorption, DLS, and zeta potential measurements to identify structural, textural, morphological, and surface-charge features relevant to Cd(ii) uptake. The adsorption performance was systematically evaluated as a function of solution pH, sorbent dose, contact time, initial Cd(ii) concentration, and temperature. Kinetic, isotherm, and thermodynamic models were applied to clarify the adsorption rate behavior, equilibrium characteristics, favorability, and energetic nature of the process. In addition, desorption using mineral acids and application to synthetic multimetal wastewater were investigated to assess the regeneration potential and practical applicability of the sorbent under more complex aqueous conditions. The study therefore aims not only to report Cd(ii) removal efficiency, but also to establish how the surface chemistry and porous structure of UA-NARB govern its adsorption behavior and environmental relevance.

## 2. Materials and methods

### 2.1. Materials and chemicals

Raw rice husk was collected from a local agricultural source and used as the lignocellulosic precursor for UA-NARB preparation. Before carbonization, the rice husk was washed successively with tap water and double-distilled water to remove dust, surface-adhered impurities, and water-soluble residues, then dried. Urea (CO(NH_2_)_2_, ≥99%, Sigma-Aldrich, USA) and acrylamide (C_3_H_5_NO, ≥99%, Sigma-Aldrich, USA) were employed as nitrogen-containing precursors for *in situ* nitrogen functionalization, while potassium carbonate (K_2_CO_3_, ≥99%, Sigma-Aldrich, USA) served as the chemical activating agent. Cadmium sulfate (CdSO_4_·*x*H_2_O, ≥99%, Sigma-Aldrich, USA) was used as the Cd(ii) source. A 1000 mg L^−1^ Cd(ii) stock solution was prepared by dissolving an accurately weighed amount of cadmium sulfate in double-distilled water, and the required working solutions were freshly obtained by dilution before each adsorption experiment. The solution pH was adjusted using standardized HCl or NaOH solutions (0.5 M). Analytical-grade HCl, H_2_SO_4_, and HNO_3_ were used as eluents in the desorption experiments. Unless otherwise stated, all chemicals were used as received without further purification, and double-distilled water was used throughout all preparation, washing, adsorption, dilution, and desorption procedures.

### 2.2. Preparation of nitrogen-functionalized rice husk biochar sorbent

The urea/acrylamide-assisted nitrogen-functionalized activated rice husk biochar was prepared through a two-step thermochemical route involving preliminary carbonization of rice husk followed by simultaneous K_2_CO_3_-assisted activation and *in situ* nitrogen functionalization, as schematically illustrated in Scheme 1. The preparation strategy was adapted from reported approaches for thermochemically engineered N-doped biochars and alkali/carbonate-activated porous carbons, in which nitrogen precursors enrich surface functionality while carbonate activation improves pore accessibility.^22,26^ Briefly, raw rice husk was thoroughly washed with tap water followed by double-distilled water to remove adhering dust, water-soluble impurities, and loosely attached residues. The washed biomass was oven-dried at 105 °C for 24 h and then subjected to slow pyrolysis at 300 °C for 2 h under an N_2_ atmosphere to obtain the primary rice husk biochar. The obtained primary biochar was lightly ground, sieved to a particle size below 250 µm, and homogeneously mixed with K_2_CO_3_, urea, and acrylamide at a mass ratio of primary biochar : K_2_CO_3_ : urea : acrylamide = 1 : 2: 1 : 1 (w/w/w/w). In this formulation, K_2_CO_3_ served as the chemical activating agent, while urea and acrylamide were used as nitrogen-containing precursors.^22,26^ The impregnated mixture was thermally treated at 600 °C for 2 h at a heating rate of approximately 10 °C min^−1^ under an N_2_ atmosphere. After cooling naturally to room temperature, the activated product was repeatedly washed with hot double-distilled water followed by 0.1 M HCl to remove residual potassium-containing species, unreacted activating agent, and soluble inorganic impurities. The solid was then rinsed several times with double-distilled water until the filtrate reached near-neutral pH, dried at 80 °C, lightly ground, and stored in airtight containers for further characterization and adsorption experiments.^22,26^ The final sorbent was designated UA-NARB, referring to urea/acrylamide-assisted nitrogen-functionalized activated rice husk biochar.

**Scheme 1:**
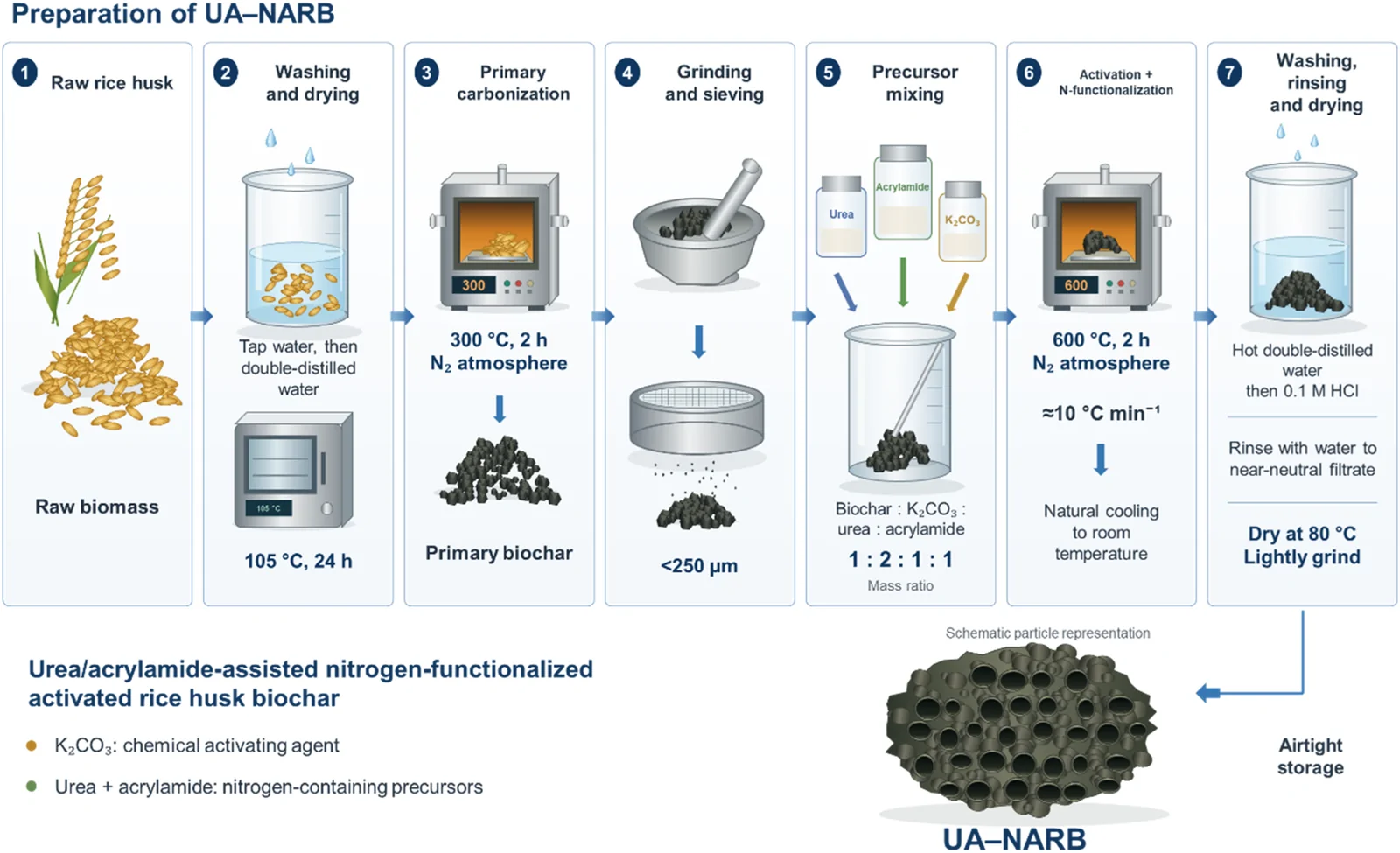
Schematic preparation route of UA-NARB.

### 2.3. Characterization of UA-NARB

The physicochemical properties of UA-NARB were examined using complementary characterization techniques to define its structural order, surface chemistry, morphology, porosity, aqueous dispersion behavior, and surface charge. The crystalline and semi-crystalline features of the sorbent were analyzed by X-ray diffraction (XRD) using a PANalytical X’Pert PRO diffractometer (Netherlands) equipped with Cu Kα radiation (*λ* = 1.5406 Å). XRD patterns were collected over a 2*θ* range of 5–80° with a step size of 0.02°, enabling assessment of the carbonaceous phase and mineral components associated with the rice husk precursor, particularly silica-related residues. Fourier-transform infrared (FTIR) spectroscopy was employed to identify the main surface functional groups and bonding environments. FTIR spectra were recorded using a PerkinElmer Spectrum One spectrometer (USA) over the range of 4000–400 cm^−1^ at a resolution of 4 cm^−1^. The surface morphology of UA-NARB was investigated by scanning electron microscopy (SEM) using a JEOL JSM microscope operated at an accelerating voltage of 20 kV. Before imaging, the sample was coated with a thin gold layer to improve electrical conductivity. SEM observations were used to examine particle texture, surface roughness, cracks, cavities, and pore openings generated by the thermochemical activation process. The textural properties of UA-NARB were determined from N_2_ adsorption–desorption isotherms measured at 77 K using a Quantachrome NOVA touch 4LX analyzer. Prior to analysis, the sample was degassed under vacuum at 200 °C for 6 h to remove adsorbed moisture and volatile species. The specific surface area was calculated using the Brunauer–Emmett–Teller (BET) method, while pore-volume and pore-size distributions were evaluated using the Barrett–Joyner–Halenda (BJH) and density functional theory (DFT) approaches. The total pore volume was estimated near saturation at *P*/*P*_0_ ≈ 0.995. The aqueous dispersion behavior and electrokinetic surface properties of UA-NARB were further examined using dynamic light scattering (DLS) and zeta potential measurements on a particle sizing systems instrument (Santa Barbara, USA). DLS measurements were conducted using a 632.8 nm laser to determine the hydrodynamic particle-size distribution of UA-NARB dispersed in water. For zeta-potential measurements, 0.2 g of UA-NARB was dispersed in 5 mL of Milli-Q water and probe-sonicated for 1 min at 70% amplitude. The zeta potential was then measured by electrophoretic light scattering at 25 °C under an applied electric field of 3.0 V cm^−1^, with all measurements conducted under identical conditions. Together, these techniques provided an integrated description of the structure, surface functionality, morphology, porosity, and colloidal behavior of UA-NARB, supporting subsequent interpretation of its Cd(ii) adsorption performance.

### 2.4. Cd(ii) adsorption experiments and data analysis

#### 2.4.1. Batch Cd(ii) adsorption experiments

Batch experiments were conducted to evaluate the Cd(ii) removal performance of UA-NARB under different operating conditions. Unless otherwise stated, adsorption tests were performed at a sorbent dose of 2.0 g L^−1^ using 20 mg L^−1^ Cd(ii) solution. The suspensions were agitated at 150 rpm for the selected contact time, after which the solid and liquid phases were separated by filtration through 0.22 µm membrane filters. The residual Cd(ii) concentration in the filtrate was determined by atomic absorption spectroscopy (AAS). All adsorption measurements were conducted in triplicate, and the mean values were reported when the relative standard deviation did not exceed 5%. The effect of solution pH was examined over the pH range of 2–8 using 20 mg L^−1^ Cd(ii), 2.0 g L^−1^ UA-NARB, and a solution volume of 1.0 L. The initial pH was adjusted before sorbent addition using dilute HCl or NaOH solutions. For the pH-effect experiments, the dissolved Cd(ii) concentration was measured after pH adjustment and before UA-NARB addition to verify that pH adjustment alone did not decrease *C*_0_. Initial and final pH values were also recorded after equilibration, with the observed variation remaining within ± 0.05 pH unit over the investigated range. The influence of sorbent dose was evaluated by varying the UA-NARB loading from 0.5 to 2.5 g L^−1^ at an initial Cd(ii) concentration of 20 mg L^−1^. Contact-time experiments were carried out over 5–600 min to assess the adsorption rate and equilibrium behavior. Equilibrium isotherm experiments were performed using initial Cd(ii) concentrations of 5–50 mg L^−1^ under the selected sorbent dose and contact conditions. Temperature-dependent adsorption experiments were conducted at 25, 30, 40, and 50 °C to evaluate the thermodynamic behavior of Cd(ii) adsorption onto UA-NARB. The Cd(ii) removal efficiency, equilibrium adsorption capacity, and distribution coefficient were calculated using eqn (1)–(3) respectively:1
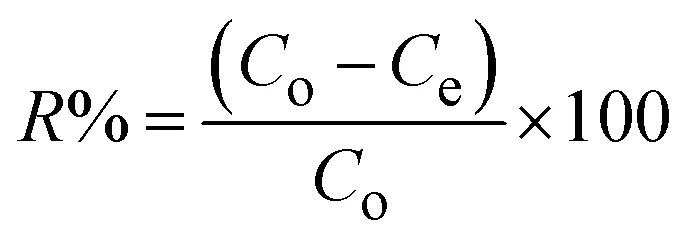
2
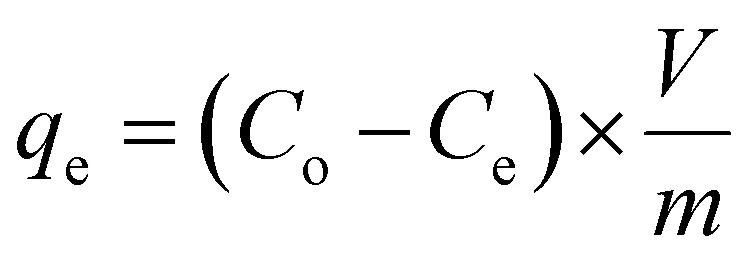
3
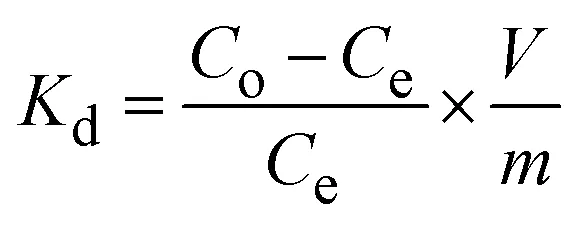
where *R* is the Cd(ii) removal efficiency (%), *q*_e_ is the equilibrium adsorption capacity (mg g^−1^), *K*_d_ is the distribution coefficient (L g^−1^), *C*_o_ and *C*_e_ are the initial and equilibrium Cd(ii) concentrations (mg L^−1^), respectively, *V* is the solution volume (L), and *m* is the mass of UA-NARB (g).

The desorption behavior of Cd(ii)-loaded UA-NARB was investigated using mineral acid eluents to assess the reversibility of Cd(ii) binding and the regeneration potential of the sorbent. After adsorption, the loaded sorbent was separated by filtration, gently washed with distilled water to remove weakly retained solution-phase Cd(ii), and then contacted with HCl, H_2_SO_4_, or HNO_3_ under the selected desorption conditions. The concentration of Cd(ii) released into the eluent was measured by AAS. The practical applicability of UA-NARB was further assessed using a synthetic multimetal wastewater prepared to simulate the composition of wastewater supplied from wastewater treatment stations. The solution contained Cd(ii), Cu(ii), Zn(ii), Mn(ii), Fe(iii), and Al(iii) at initial concentrations of 1.0, 10.3, 11.0, 12.0, 25.0, and 8.0 mg L^−1^, respectively. Treatment was conducted under the selected adsorption conditions, and the residual metal concentrations were measured by AAS. This experiment was designed to evaluate UA-NARB performance in a competitive multicomponent matrix, where coexisting ions and dissolved species may influence Cd(ii) uptake compared with simplified single-metal systems.

#### 2.4.2. Modeling and fitting analysis

The contact-time data were fitted using pseudo-first-order, pseudo-second-order, Weber–Morris intraparticle diffusion, and Bangham models to clarify the adsorption-rate behavior and the possible contribution of diffusion during Cd(ii) uptake onto UA-NARB.^27–30^ The pseudo-first-order model was used to evaluate adsorption behavior controlled by the difference between equilibrium and time-dependent uptake, whereas the pseudo-second-order model was applied to assess the dependence of Cd(ii) uptake on active-site availability and surface-interaction-controlled adsorption. The Weber–Morris intraparticle diffusion model was used to examine internal diffusion, while Bangham's model was applied to further assess pore-diffusion effects. The nonlinear equations of the applied kinetic models are summarized in Table S1. Equilibrium adsorption data obtained at different initial Cd(ii) concentrations were fitted using Langmuir, Freundlich, Dubinin–Radushkevich, and Sips isotherm models.^28–32^ The Langmuir model was applied to evaluate monolayer adsorption onto a finite number of energetically comparable sites, while the Freundlich model was used to describe adsorption on heterogeneous surfaces. The Dubinin–Radushkevich model was used to estimate the apparent adsorption energy, and the Sips model was applied as a combined Langmuir–Freundlich expression suitable for systems showing finite saturation capacity with a degree of surface heterogeneity. The favorability of Langmuir adsorption was further evaluated using the dimensionless separation factor *R*_L_. The nonlinear equations of the applied isotherm models and the *R*_L_ expression are provided in Table S1. Thermodynamic parameters were calculated from temperature-dependent Cd(ii) adsorption experiments.^33,34^ The standard Gibbs free energy change (Δ*G*∘) was calculated from the thermodynamic equilibrium constant, while the standard enthalpy (Δ*H*∘) and entropy (Δ*S*∘) changes were estimated using the van't Hoff relationship. The corresponding equations are listed in Table S1. These parameters were used to evaluate the spontaneity, heat effect, and interfacial randomness associated with Cd(ii) adsorption onto UA-NARB.

The kinetic and isotherm models were evaluated using both statistical fitting and physical consistency of the estimated parameters. The coefficient of determination (*R*^2^) was used to assess the agreement between experimental and calculated values, while the chi-square error function (*χ*^2^) was applied to quantify the deviation between measured and predicted adsorption capacities.^30–32^ The corresponding equations are summarized in Table S1. The most suitable model was selected based on higher *R*^2^, lower *χ*^2^, agreement between experimental and calculated adsorption capacities, and consistency of the estimated parameters with the physicochemical properties of UA-NARB.

## 3. Results & discussion

### 3.1. Characterization of UA-NARB

The physicochemical properties of UA-NARB were systematically examined to establish the structural and surface features governing Cd(ii) adsorption. XRD and FTIR analyses were first used to identify the crystalline/amorphous domains and surface functional groups, while SEM and N_2_ adsorption–desorption measurements were applied to evaluate morphology and porous texture. DLS and zeta potential analyses were further conducted to assess the aqueous dispersion behavior and electrokinetic surface charge of the sorbent, which are directly relevant to Cd(ii) transport and surface interaction in aqueous suspension.

#### 3.1.1. Structural and surface chemical features

The structural and surface-chemical features of UA-NARB were first examined by XRD and FTIR to identify the main crystalline/amorphous domains and surface functionalities relevant to Cd(ii) adsorption. As shown in Fig. 1a, the XRD pattern of UA-NARB displays a weak and poorly defined carbon-related background across the investigated 2*θ* range, indicating that the sorbent is dominated by a largely disordered carbonaceous framework rather than a highly graphitized structure. This behavior is typical of biochars and biomass-derived carbons prepared from lignocellulosic precursors, where thermochemical conversion generally produces turbostratic or amorphous carbon domains with limited long-range ordering.^5,18^ From an adsorption perspective, this structural disorder is advantageous because it can provide edge defects, heterogeneous surface environments, and accessible reactive domains that may facilitate Cd(ii) interaction directly or through adjacent oxygenated, nitrogen-containing, and mineral-associated sites.^5,18^ In addition to the disordered carbon matrix, distinct diffraction peaks are observed at approximately 31.45°, 36.99°, 44.98°, 55.86°, 59.50°, and 65.38° (2*θ*) (Fig. 1a), indicating the presence of crystalline inorganic domains retained or formed during K_2_CO_3_-assisted activation and post-treatment. The coexistence of amorphous carbon and mineral-rich domains is important for Cd(ii) adsorption because defect-rich carbon edges, mineral oxygen sites, silanol-type groups, and carbonate/carboxylate-associated species may collectively provide diverse interaction centers for metal-ion binding.^5,34^

**Fig. 1:**
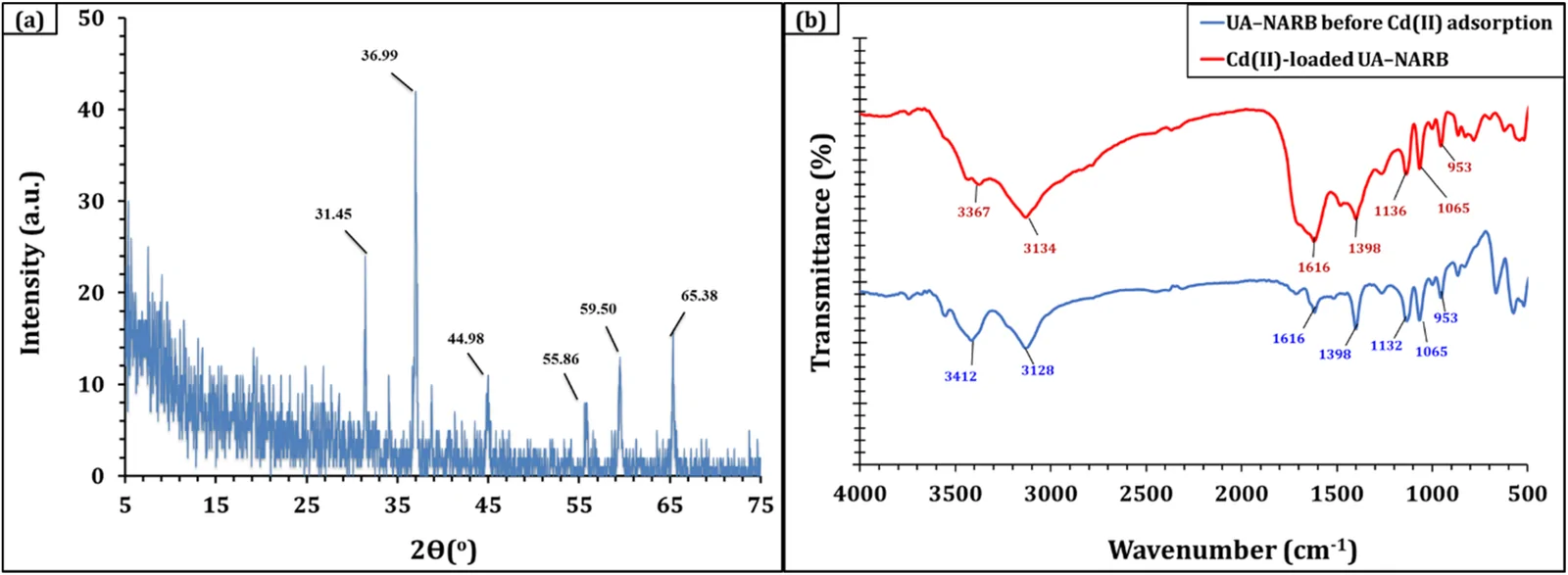
Structural characterization of UA-NARB: (a) XRD pattern with principal diffraction peaks and (b) FTIR spectra before and after Cd(ii) adsorption.

The FTIR spectra further confirm the chemically heterogeneous surface of UA-NARB (Fig. 1b). Before Cd(ii) adsorption, the principal bands occur at approximately 3412, 3128, 1616, 1398, 1132, 1065, and 953 cm^−1^. The broad absorption band centered around 3412 cm^−1^ is mainly assigned to O–H stretching vibrations of hydroxyl groups and physically adsorbed water, with a possible contribution from N–H vibrations associated with urea/acrylamide-derived nitrogen function.^4,18,19^These polar groups are relevant to Cd(ii) adsorption because hydroxyl, amino/amide-like, and other proton-active sites can influence surface charge development and provide electron-donating centers after partial deprotonation under suitable pH conditions.^6,25,35^ The weak feature around 3128 cm^−^ is consistent with overlapping O–H/N–H-related vibrations after carbonization, activation, and washing. In the mid-wavenumber region, the band near 1616 cm^−^ can be attributed to overlapping contributions from C=O stretching of carbonyl/carboxyl-type groups, aromatic C=C vibrations, and conjugated carbon structures formed during thermochemical conversion (Fig. 1b).^6,18,35^ Such groups are important because carboxyl, carbonyl, and related oxygenated sites commonly participate in Cd(ii) interaction through electrostatic attraction and surface complexation, particularly when the sorbent surface becomes partially deprotonated.^5,6^ Meanwhile, the absorption bands located between approximately 1400 and 1000 cm^−1^, including those near 1398, 1132, and 1065 cm^−1^, are consistent with overlapping C–N, C–O, C–O–C, and Si–O-related vibrations, reflecting the combined effects of nitrogen precursor decomposition, oxygenated carbon formation, and the mineral-rich nature of rice husk-derived biochar.^6,18,19,25^ The intense fingerprint bands near 953 cm^−1^ are mainly related to Si–O–Si, Si–O, metal–oxygen, and mineral-associated vibrations inherited from the rice husk precursor or developed during K_2_CO_3_-assisted activation (Fig. 1b). For comparison, the FTIR spectrum of Cd(ii)-loaded UA-NARB is also presented in Fig. 1b. After adsorption, the main O–H/N–H-related band shifted from approximately 3412 to 3367 cm^−1^, accompanied by smaller changes around 3130, 1616, 1398, 1132–1136, 1065, and 953 cm^−1^. These spectral changes indicate modification of the local chemical environment of surface functional groups following Cd(ii) uptake. The relationship between these changes and the proposed Cd(ii) interaction pathways is discussed in detail in the mechanism section. These features agree with the XRD results, confirming that UA-NARB is a hybrid carbon–mineral sorbent rather than a purely carbonaceous material. The coexistence of O-/N-containing functional groups with mineral-associated oxygen sites provides diverse chemically active domains that can contribute to Cd(ii) uptake. Overall, the XRD and FTIR results demonstrate that UA-NARB possesses a structurally disordered and chemically functionalized surface, providing a rational basis for the subsequent adsorption behavior.^5,6,25^

#### 3.1.2. Morphological and surface properties of UA-NARB

The morphological features of UA-NARB were examined by SEM to clarify how carbonization and K_2_CO_3_-assisted activation affected the surface texture and accessibility of the prepared sorbent. As shown in Fig. 2a, UA-NARB exhibits an irregular and fragmented morphology composed of rough carbonaceous particles, plate-like fragments, cavities, cracks, and small surface deposits. This texture is consistent with the combined effects of biomass carbonization and chemical activation, where thermal decomposition, volatile release, and carbonate-assisted etching can disrupt the original lignocellulosic structure and generate a rougher, more open surface.^6,18,22^ From an adsorption viewpoint, this morphology is favorable because exposed edges, surface defects, cracks, and cavities can increase contact between the Cd(ii)-bearing solution and the sorbent surface, while also facilitating access of hydrated Cd(ii) species toward internal adsorption domains. The brighter, finely dispersed surface features are consistent with the presence of mineral-associated domains retained from the rice husk precursor or developed during activation, in agreement with the XRD and FTIR evidence for mineral-related and Si–O-containing structures. These carbonaceous and mineral-associated domains may jointly provide accessible surface sites for Cd(ii) interaction, supporting the role of UA-NARB as a structurally heterogeneous carbon–mineral sorbent.^5,6^

**Fig. 2:**
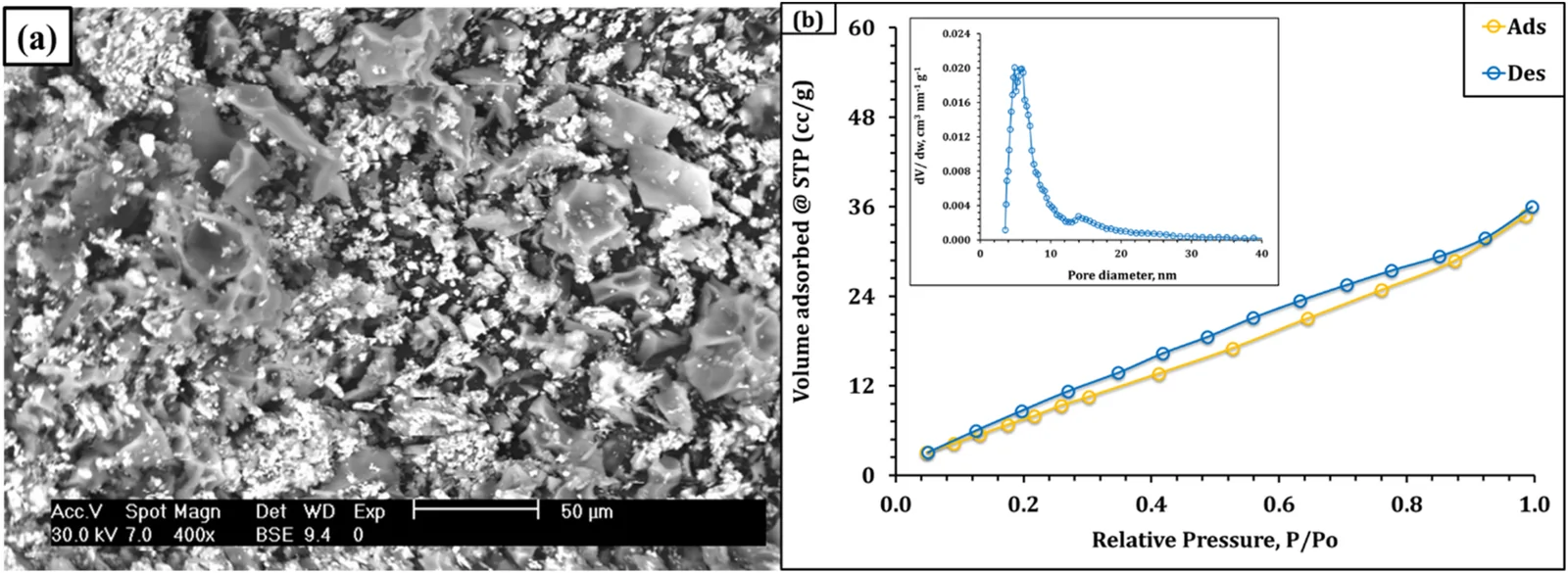
Morphological and textural characterization of UA-NARB: (a) SEM image and (b) N_2_ adsorption–desorption isotherm with pore-size distribution inset.

The porous-textural properties of UA-NARB were evaluated by N_2_ adsorption–desorption analysis to assess the effect of K_2_CO_3_-assisted activation on pore development. As shown in Fig. 2b, the isotherm exhibits a gradual increase in adsorbed N_2_ volume over the studied relative pressure range, accompanied by a narrow hysteresis loop between the adsorption and desorption branches. This profile indicates the presence of accessible mesoporous domains and interparticle voids rather than a purely microporous structure.^6,22^ The increased N_2_ uptake at higher relative pressure further suggests the contribution of mesopore filling and larger interparticle spaces, which can facilitate liquid-phase transport during Cd(ii) adsorption. UA-NARB exhibited a BET surface area of 40.9 m^2^ g^−1^ and a total pore volume of 0.055 cm^3^ g^−1^ (Table 1), indicating a moderately developed porous structure after carbonate-assisted activation. Although the surface area is not exceptionally high, it remains adsorption-relevant when combined with favorable surface chemistry, negative surface charge, and O-/N-containing functional groups. This agrees with the concept that heavy-metal adsorption by functionalized biochars is governed not only by total surface area, but also by the accessibility and chemical nature of the active binding sites.^5,6^ The pore-size parameters further confirm the mesoporous character of UA-NARB. BJH analysis gave a pore volume of 0.047 cm^3^ g^−1^ and a dominant pore radius of 1.91 nm, corresponding to an approximate pore diameter of 3.82 nm (Table 1). These values fall within the mesoporous range, which is advantageous for aqueous Cd(ii) adsorption because mesopores can reduce diffusion resistance and improve access of hydrated metal ions to internal adsorption sites.^6,22^ The pore-size distribution inset in Fig. 2b also shows a dominant low-mesopore contribution with a gradually decreasing tail toward larger pore sizes, indicating the coexistence of small mesopores and broader transport voids. Overall, the textural results show that UA-NARB possesses a moderate but accessible porous network that supports diffusion-assisted Cd(ii) transport, while adsorption strength is expected to depend mainly on the available O-/N-containing and mineral-associated surface sites.

**Table 1:** Textural, dispersion, and surface-charge properties of UA-NARB

Property	Value
Surface area	40.9 m^2^ g^−1^
Total pore volume	0.055 cm^3^ g^−1^
Pore radius	1.91 nm
Pore diameter	3.82 nm
DLS mean diameter	1040.8 nm
Zeta potential	−16.54 mV

The aqueous dispersion behavior and electrokinetic surface properties of UA-NARB were evaluated by DLS and zeta potential measurements to clarify its behavior under suspension-based Cd(ii) adsorption conditions. The intensity-weighted DLS analysis showed a mean hydrodynamic diameter of 1040.8 nm, with 25%, 50%, 75%, 80%, 90%, and 99% of the particle distribution below 410.1, 725.2, 1295.9, 1497.3, 2189.6, and 5286.4 nm, respectively (Table 1 and Fig. S1a). The relatively high polydispersity index of 0.76 indicates that UA-NARB forms a heterogeneous aqueous suspension rather than a narrowly dispersed particle population. This behavior is consistent with the irregular SEM morphology and can be attributed to the coexistence of porous carbon fragments, mineral-associated domains, and partially aggregated particles.^26,36^ Zeta potential analysis gave an average value of −16.54 mV at 23 °C (Table 1 and Fig. S1b), indicating that UA-NARB possesses a moderately negative surface charge under the measurement conditions. This charge favors the attraction of Cd(ii) cations, particularly as solution pH increases and H^+^ competition decreases.^6,8^ However, because the zeta potential is moderate, electrostatic attraction should be considered a contributing pathway rather than the sole mechanism of Cd(ii) uptake. Accordingly, the DLS and zeta potential results support the interpretation that UA-NARB behaves as a heterogeneous, negatively charged sorbent with surface properties favorable for Cd(ii) interaction in aqueous suspension.

Overall, the characterization results demonstrate that UA-NARB possesses a heterogeneous carbon–mineral structure with disordered carbon domains, retained inorganic/mineral phases, oxygen- and nitrogen-containing surface functionalities, accessible mesoporosity, and a moderately negative surface charge. These features collectively support Cd(ii) adsorption by providing transport pathways and chemically active interaction sites. Therefore, the adsorption behavior of UA-NARB should be interpreted as the combined outcome of pore accessibility, surface charge, and functional/mineral–site interactions rather than as a process controlled by surface area alone.

### 3.2. Effect of adsorption parameters on Cd(ii) removal by UA-NARB

#### 3.2.1. Effect of solution pH and sorbent dose

Solution pH is a critical parameter in Cd(ii) adsorption because it controls both metal speciation in solution and the protonation state of the sorbent surface. Accordingly, the adsorption performance of UA-NARB was evaluated over the pH range of 2–8 using 20 mg L^−1^ Cd(ii) and 2.0 g L^−1^ sorbent dose. As shown in Fig. 3a, Cd(ii) removal increased markedly from 49.8% at pH 2 to 66.7% at pH 3 and 85.9% at pH 4, then reached 91.8 and 94.5% at pH 5 and 6, respectively. Further increase in pH produced only a marginal improvement, with removal reaching 95.1% at pH 8. The adsorption capacity followed the same trend, increasing from 5.0 mg g^−1^ at pH 2 to approximately 9.5 mg g^−1^ at pH 6–8. The low uptake under strongly acidic conditions is mainly attributed to competition between H^+^ and Cd(ii) for available adsorption sites and protonation of surface functionalities, which reduces the density of negatively charged or electron-donating sites.^6,8,25^ As pH increases, progressive deprotonation increases the availability of favorable surface sites for Cd(ii) interaction.^7,8,25^ consistent with the moderately negative zeta potential of UA-NARB and the FTIR-identified polar surface functionalities (Table 1 and Fig. 1b). Because both Cd(ii) aqueous speciation and sorbent surface chemistry are pH-dependent, Cd(ii) speciation was evaluated using MEDUSA/HYDRA software (Fig. S4). The analysis showed that dissolved Cd^2+^ remains the predominant species throughout the investigated range up to pH 8, whereas Cd(OH)_2_(s) becomes increasingly important above this range. The initial and final pH values also remained closely matched (Table S2), with a maximum deviation of 0.05 pH unit. Together, these results indicate that the pronounced increase in Cd(ii) removal up to pH 6 is primarily associated with favorable pH-dependent surface interactions rather than bulk Cd(OH)_2_ precipitation. The near-plateau between pH 6 and 8 indicates limited additional benefit from further pH increase. Therefore, pH 6 was selected for subsequent experiments because it provides high Cd(ii) removal under near-neutral conditions while remaining below the pH range where Cd(OH)_2_ precipitation becomes significant.

**Fig. 3:**
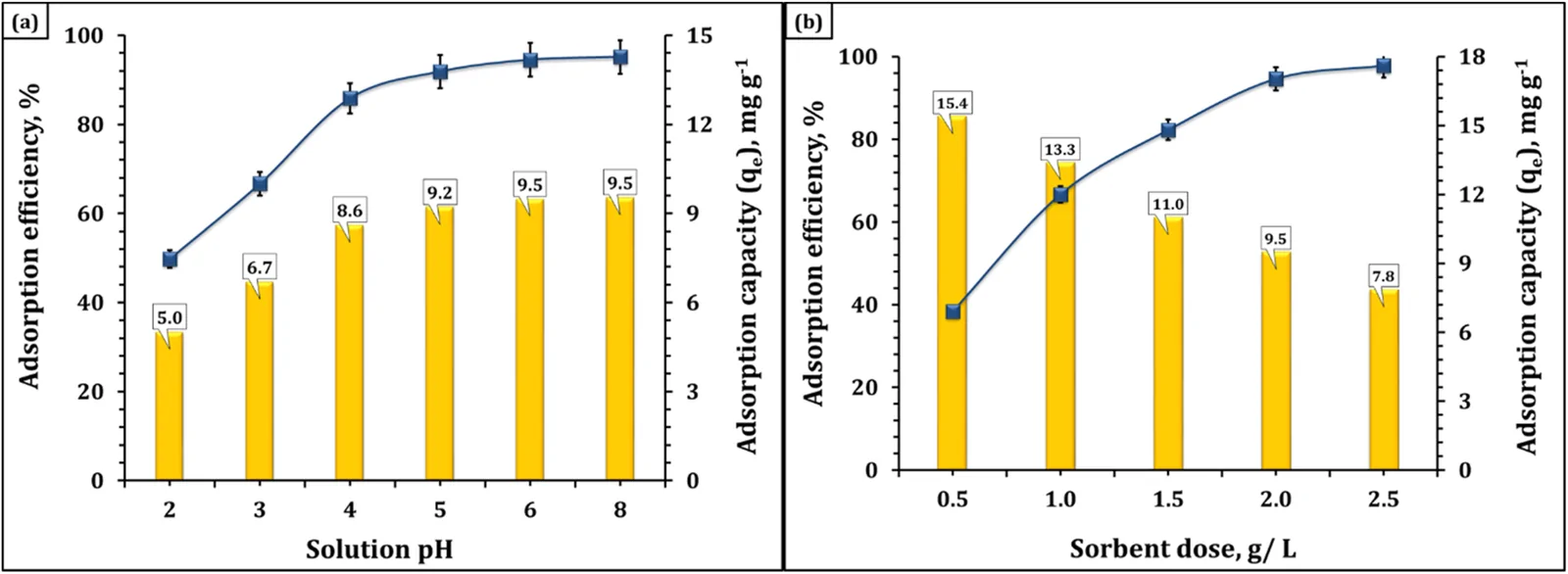
Effect of operating parameters on Cd(ii) adsorption by UA-NARB: (a) effect of solution pH on removal efficiency and adsorption capacity using 20 mg L^−1^ Cd(ii) and 2.0 g L^−1^ sorbent dose; (b) effect of UA-NARB dose on Cd(ii) removal efficiency, adsorption capacity, and distribution coefficient at 20 mg L^−1^ Cd(ii).

The effect of sorbent dose was examined to identify the UA-NARB loading that provides high Cd(ii) removal while maintaining efficient sorbent utilization. The dose was varied from 0.5 to 2.5 g L^−1^ at an initial Cd(ii) concentration of 20 mg L^−1^. As shown in Fig. 3b, the removal efficiency increased progressively from 38.4% at 0.5 g L^−1^ to 66.7% at 1.0 g L^−1^ and 82.2% at 1.5 g L^−1^, reaching 94.6% at 2.0 g L^−1^ and 97.8% at 2.5 g L^−1^. This improvement is attributed to the greater number of available adsorption sites and accessible surface domains introduced at higher sorbent loading, which increases the probability of Cd(ii) capture.^6,35,37^ The corresponding increase in log K_d_ from 0.1 to 1.2 further confirms the enhanced partitioning of Cd(ii) from the aqueous phase to the solid phase as the UA-NARB dose increased. In contrast, the equilibrium adsorption capacity decreased from 15.4 mg g^−1^ at 0.5 g L^−1^ to 13.3, 11.0, 9.5, and 7.8 mg g^−1^ at 1.0, 1.5, 2.0, and 2.5 g L^−1^, respectively. This inverse relationship is commonly observed when the initial metal concentration is fixed. At low sorbent dose, the available sites are more fully occupied, resulting in higher Cd(ii) loading per gram. At higher doses, the number of binding sites exceeds the amount of Cd(ii) available in solution, leaving more sites unsaturated and lowering the calculated *q*_e_.^6,35,37^ Thus, removal efficiency and adsorption capacity should be interpreted together to avoid overestimating performance based only on percentage removal. Although 2.5 g L^−1^ produced the highest removal efficiency, the improvement relative to 2.0 g L^−1^ was limited, increasing from 94.6 to 97.8%, while *q*_e_ decreased from 9.5 to 7.8 mg g^−1^. Therefore, 2.0 g L^−1^ was selected as the working dose because it achieved high Cd(ii) removal while maintaining better sorbent utilization. This dose was subsequently used in the kinetic, isotherm, and thermodynamic experiments to ensure consistent evaluation under balanced operating conditions.

#### 3.2.2. Effect of contact time and adsorption kinetics

The effect of contact time was investigated to evaluate the rate of Cd(ii) uptake by UA-NARB and determine the time required to approach equilibrium. The experiment was performed using 20 mg L^−1^ Cd(ii), 2.0 g L^−1^ UA-NARB, and 1.0 L solution volume. As shown in Fig. 4a, Cd(ii) removal increased sharply during the initial period, rising from 20.0% after 5 min to 40.0, 58.4, and 74.0% after 15, 30, and 60 min, respectively. The corresponding adsorption capacity increased from 2.0 to 7.4 mg g^−1^ within the first 60 min, confirming that a substantial fraction of Cd(ii) removal occurred during the early contact stage. Thereafter, the rate gradually declined, with removal increasing from 80.5% at 120 min to 87.0% at 240 min, before approaching 95.0–95.5% at 480–600 min. The adsorption capacity followed the same trend, reaching a near-equilibrium value of 9.5–9.6 mg g^−1^ at prolonged contact time. The fast initial removal can be attributed to the high availability of accessible binding sites on the external surface of UA-NARB, including O-/N-containing groups, negatively charged domains, and mineral-associated oxygen sites. At this stage, the large concentration gradient between the solution and sorbent surface promotes Cd(ii) transfer toward active sites.^6,7,38,39^ With increasing contact time, readily accessible sites become progressively occupied, and Cd(ii) migration into less accessible pores and internal surface domains becomes more important. This transition accounts for the slower later-stage adsorption and gradual approach to equilibrium. Overall, the contact-time profile suggests a multistep process involving fast external surface interaction followed by diffusion-assisted uptake within the porous and heterogeneous biochar structure.

**Fig. 4:**
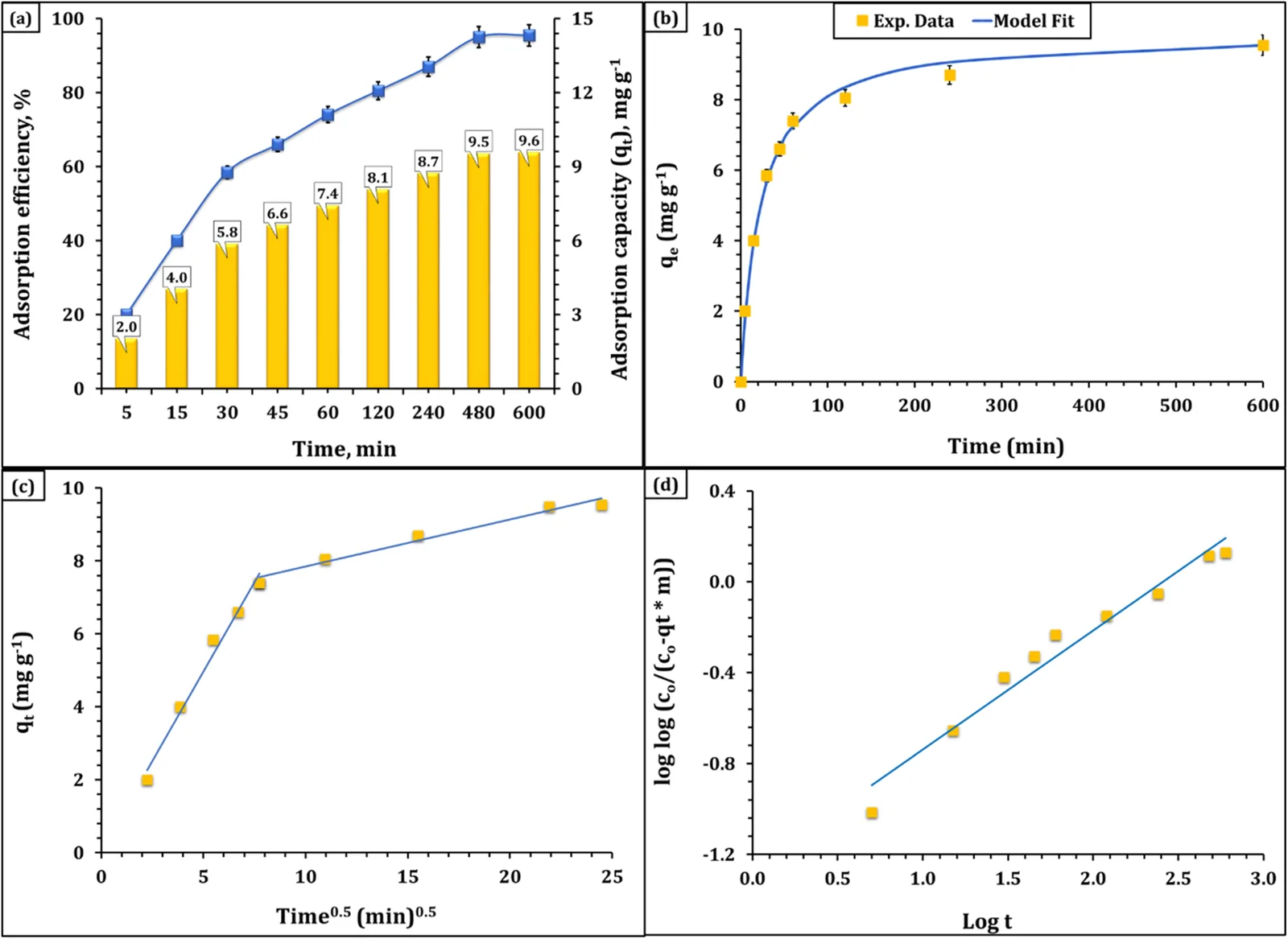
Effect of contact time and adsorption kinetic modeling for Cd(ii) uptake by UA-NARB: (a) Time-dependent removal efficiency and adsorption capacity at 20 mg L^−1^ Cd(ii) and 2.0 g L^−1^ sorbent dose; (b) Pseudo-first-order and pseudo-second-order kinetic fitting; (c) Weber–Morris intraparticle diffusion plot showing two diffusion stages; (d) Bangham model fitting.

To further interpret the adsorption-rate behavior, the contact-time data were fitted using pseudo-first-order and pseudo-second-order kinetic models (Fig. 4b and Table 2).^27–30^ The pseudo-first-order model gave a calculated adsorption capacity of 8.7 mg g^−1^, a rate constant of 0.036 min^−1^, *R*^2^ = 0.96, and *χ*^2^ = 0.50. In comparison, the pseudo-second-order model showed a better fit, with *q*_2_ = 9.9 mg g^−1^, *k*_2_ = 0.005 g mg^−1^ min^−1^, initial adsorption rate *h* = 0.4 mg g^−1^ min^−1^, half-adsorption time *t*_1_/_2_ = 22.1 min, *R*^2^ = 0.99, and *χ*^2^ = 0.05. The superiority of the pseudo-second-order model is supported by its higher *R*^2^, lower *χ*^2^, and closer agreement between the calculated *q*_2_ value and the experimental equilibrium capacity of approximately 9.6 mg g^−1^. The better agreement of the pseudo-second-order model indicates that the adsorption rate depends strongly on the extent of site occupation and on interactions between Cd(ii) ions and available surface functionalities. When considered together with the diffusion analyses, this behavior is consistent with adsorption-site occupation accompanied by diffusion-assisted transport within the porous biochar matrix. Similar pseudo-second-order behavior has been reported for Cd(ii) adsorption onto functionalized biochars and N-doped porous carbon materials,^8,35^ as well as for dye adsorption onto other engineered adsorbents.^40,41^

**Table 2:** Kinetic model parameters for Cd(ii) adsorption onto UA-NARB

Pseudo first-order model	Experimental*q*_e_ (mg g^−1^)	9.6
	*q*_1_ (mg g^−1^)	8.7
	*k*_1_ (min^−1^)	0.036
	*R* ^2^	0.96
	*X* ^2^	0.50
Pseudo second-order model	Experimental*q*_e_ (mg g^−1^)	9.6
	*q*_2_ (mg g^−1^)	9.9
	*k*_2_ (g mg^−1^ min^−1^)	0.005
	*h* (mg g^−1^ min^−1^)	0.4
	*t*_1/2_ (min)	22.1
	*R* ^2^	0.99
	*X* ^2^	0.05

The Weber–Morris intraparticle diffusion model was applied to evaluate the contribution of internal diffusion to Cd(ii) uptake (Fig. 4c), and the corresponding parameters are summarized in Table S3.^27–30^ The fitting revealed two distinct diffusion stages. The first stage showed a higher intraparticle diffusion rate constant, reflecting rapid Cd(ii) transfer from the bulk solution to the external surface and easily accessible pores of UA-NARB. The second stage exhibited a lower diffusion rate constant and a larger intercept, indicating slower diffusion into internal pores and gradual occupation of less accessible sites as adsorption approached equilibrium. Importantly, the nonzero intercepts, particularly in the second stage, indicate that intraparticle diffusion was not the only rate-controlling step; boundary-layer effects and surface interactions also contributed to the overall adsorption rate.^6,7^ Bangham's model was additionally applied to assess the role of pore diffusion in Cd(ii) adsorption (Fig. 4d), with the fitting parameters also provided in Table S3. The good Bangham correlation supports the participation of pore diffusion in the adsorption process, but does not indicate that pore diffusion alone controls the overall uptake.^27–30^ This interpretation agrees with the textural characterization of UA-NARB, where mesoporous channels and interparticle voids were identified as potential transport pathways. Accordingly, the Weber–Morris and Bangham analyses indicate contributions from intraparticle and pore diffusion to the overall uptake process, with rapid initial transfer to accessible sites followed by slower diffusion-assisted transport into internal pores and less accessible surface domains.

Overall, the contact-time and kinetic results demonstrate that UA-NARB provides rapid initial Cd(ii) capture followed by gradual equilibration. The pseudo-second-order model best describes the overall kinetic profile, while Weber–Morris and Bangham analyses indicate contributions from diffusion processes without identifying them as the sole rate-limiting step. A contact time of 480 min was selected as the practical equilibrium time for subsequent isotherm and thermodynamic experiments, as Cd(ii) removal had already reached 95.0% with an adsorption capacity of 9.5 mg g^−1^, confirming an appropriate balance between adsorption efficiency and experimental practicality.

#### 3.2.3. Effect of initial Cd(ii) concentration and adsorption isotherms

The effect of initial Cd(ii) concentration was investigated to evaluate the equilibrium uptake behavior of UA-NARB and to determine how the available adsorption sites respond to increasing metal loading. The experiments were performed over an initial Cd(ii) concentration range of 5–50 mg L^−1^ using 2.0 g L^−1^ UA-NARB and 1.0 L solution volume. As shown in Fig. 5a, the Cd(ii) removal efficiency decreased progressively from 98.1% at 5 mg L^−1^ to 97.3% at 10 mg L^−1^ and 95.0% at 20 mg L^−1^, then declined more clearly to 86.0, 76.3, 70.0, 64.0, and 58.0% as the initial concentration increased to 30, 35, 40, 45, and 50 mg L^−1^, respectively. In contrast, the equilibrium adsorption capacity increased from 2.5 mg g^−1^ at 5 mg L^−1^ to 4.9, 9.5, 12.9, 13.4, 14.0, 14.4, and 14.5 mg g^−1^ at 10, 20, 30, 35, 40, 45, and 50 mg L^−1^, respectively. This opposite trend between removal efficiency and adsorption capacity is typical for adsorption systems operated at a fixed sorbent dose. At low Cd(ii) concentrations, the number of available surface sites is high relative to the amount of Cd(ii) in solution, allowing nearly complete removal. However, as the initial Cd(ii) concentration increases, the fixed number of active sites becomes progressively occupied, leading to a gradual decrease in percentage removal. Meanwhile, the increase in *q*_e_ reflects the higher concentration gradient and stronger mass-transfer driving force, which enhance Cd(ii) transport from solution to the UA-NARB surface and promote greater loading per unit sorbent mass.^6–8,35,42^ Therefore, the concentration-dependent behavior confirms that UA-NARB is highly efficient at low-to-moderate Cd(ii) concentrations, while its maximum site utilization is progressively approached at higher metal loading.

**Fig. 5:**
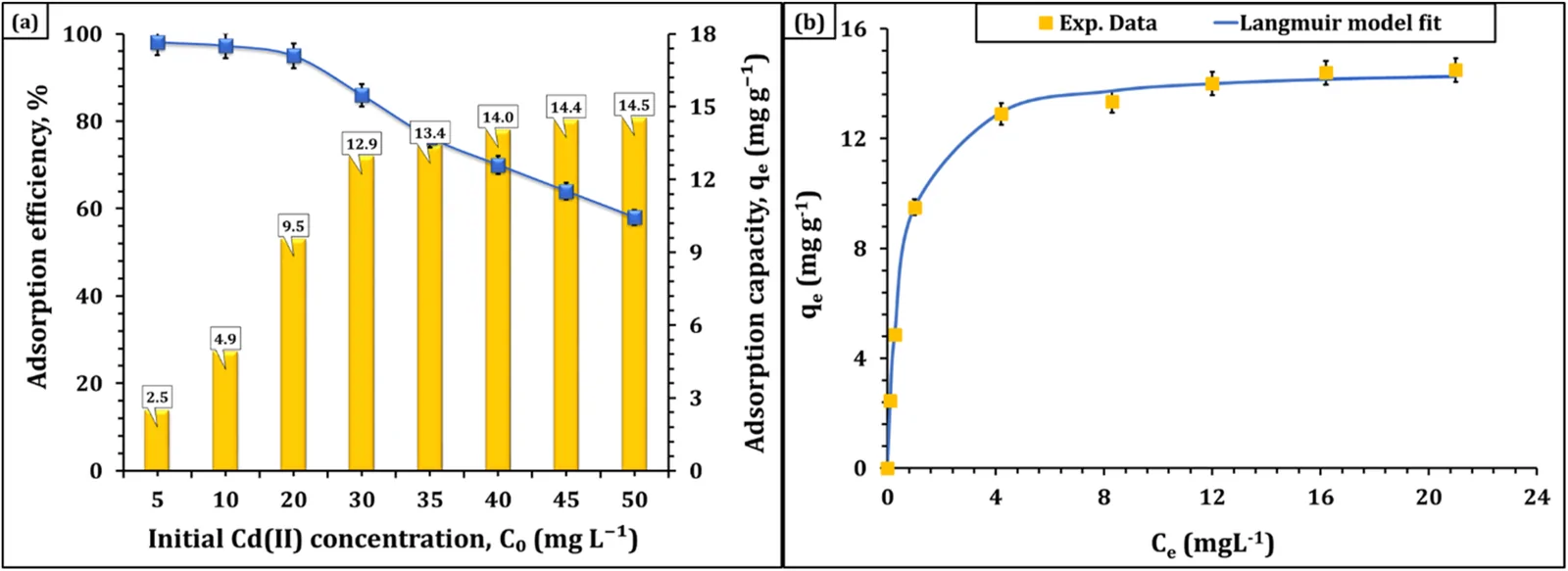
Effect of initial Cd(ii) concentration and adsorption isotherm fitting for Cd(ii) uptake by UA-NARB: (a) concentration-dependent removal efficiency and adsorption capacity using 2.0 g L^−1^ sorbent dose; (b) experimental equilibrium data with nonlinear model fitting.

To describe the equilibrium adsorption behavior, the experimental data were fitted using Langmuir, Freundlich, Dubinin–Radushkevich, and Sips isotherm models (Fig. 5b, S3 and Table 3).^28–31^ The Langmuir model showed excellent agreement with the experimental data, a fitted maximum adsorption capacity of 14.6 mg g^−1^, *K*_L_ of 1.84 L mg^−1^, *R*^2^ = 0.99, and *χ*^2^ = 0.05. The close agreement between the Langmuir *q*_m_value and the experimentally observed maximum *q*_e_ of 14.5 mg g^−1^ indicates that Cd(ii) uptake approaches a finite saturation capacity under the studied concentration range. Accordingly, the Langmuir model provides an effective mathematical description of the observed saturation behavior within the investigated concentration range.^6–8^ Comparable Langmuir-type equilibrium behavior has also been reported for the adsorption of cationic dyes onto polymeric and composite adsorbents.^43,44^ The Langmuir separation factor further confirmed the favorability of Cd(ii) adsorption onto UA-NARB. As shown in Fig. S4, the *R*_L_ values decreased from 0.098 to 0.011 as the initial Cd(ii) concentration increased from 5 to 50 mg L^−1^. Since all *R*_L_ values fall between 0 and 1, the adsorption process can be considered favorable across the entire investigated concentration range.^28–30^ The gradual decrease in *R*_L_ with increasing Cd(ii) concentration indicates greater favorability at higher initial concentration, which is consistent with the stronger concentration gradient and enhanced driving force for Cd(ii) transfer toward the sorbent surface.

**Table 3:** Isotherm model parameters for Cd(ii) adsorption onto UA-NARB

Model	Parameter	Value
Langmuir model	*q*_m_ (mg g^−1^)	14.6
	*k*_L_ (L mg^−1^)	1.84
	*R* ^2^	0.99
	*X* ^2^	0.05
Freundlich model	*n*_F_	0.22
	*k*_F_ (mg g^− 1^) (mg L^−1^)	8.2
	*R* ^2^	0.91
	*X* ^2^	2.1
D–R model	*q*_D_ (mg g^−1^)	14.0
	*B* (mol^2^ kJ^−2^)	0.07
	*E*_ad_	2.6
	*R* ^2^	0.97
	*X* ^2^	2.5
Sips model	*q*_S_ (mg g^−^ )	15.1
	*K*_S_ (L mg^−1^)	1.72
	*m*_S_	0.90
	*R* ^2^	0.99
	*X* ^2^	0.02

The Freundlich model gave 1/*n*_F_ = 0.22, *K*_F_ = 8.2, *R*^2^ = 0.91, and *χ*^2^ = 2.1 (Table 3 and Fig. S3). The 1/*n*_F_ value below unity is consistent with favorable adsorption on a heterogeneous surface.^37–39^ This agrees with the physicochemical characterization, which showed a heterogeneous carbon–mineral structure containing O-/N-functional groups, mesoporous domains, and mineral-associated oxygen sites. However, the lower *R*^2^ and higher *χ*^2^ values compared with Langmuir indicate that Freundlich is less suitable for describing the equilibrium data, suggesting that surface heterogeneity exists but does not dominate the overall adsorption behavior. The Dubinin–Radushkevich model provided *q*_D_ = 14.0 mg g^−1^, *B* = 0.07 mol^2^ kJ^−2^, *E*_ad_ = 2.6 kJ mol^−1^, *R*^2^ = 0.97, and *χ*^2^ = 2.5 (Table 3 and Fig. S3). The *q*_D_ value is close to the Langmuir *q*_m_ and the experimental maximum *q*_e_, further supporting the presence of a finite adsorption capacity for Cd(ii) on UA-NARB. The relatively low *E*_ad_ value indicates a low apparent adsorption energy within the D–R model,^28–31^ while the higher fitting error shows that this model is less representative of the equilibrium data than Langmuir and Sips. Among the tested models, the Sips model gave the best statistical fit, with *q*_S_ = 15.1 mg g^−1^, *K*_S_ = 1.72 L mg^−1^, *m*_S_ = 0.90, *R*^2^ = 0.99, and *χ*^2^ = 0.02 (Fig. 5b and Table 3). The slightly higher capacity estimated by Sips compared with Langmuir, together with the lowest *χ*^2^ value, indicates that Cd(ii) adsorption onto UA-NARB is well represented by a saturation-type model with moderate surface heterogeneity.^28–31^ The Sips heterogeneity exponent close to unity further indicates near-Langmuir behavior while accounting for moderate heterogeneity in the fitted equilibrium response.^37–39^

Overall, the initial-concentration and isotherm results demonstrate that UA-NARB exhibits favorable and saturation-limited Cd(ii) adsorption. The increase in *q*_e_ with initial concentration reflects enhanced driving force and improved site utilization, whereas the decrease in removal efficiency indicates progressive occupation of available binding sites at higher Cd(ii) loading. The Langmuir and Sips models provide the most reliable description of the equilibrium behavior, with maximum capacities of 14.6 and 15.1 mg g^−1^, respectively. These findings show that the equilibrium data are characterized by finite saturation capacity with moderate surface heterogeneity.

To further position the adsorption performance of UA-NARB, its Cd(ii) uptake was compared with selected sorbents reported in previous studies (Table 4). The Langmuir capacity of UA-NARB reached 14.6 mg g^−1^ at pH 6 and 25 °C over an initial Cd(ii) concentration range of 5–50 mg L^−1^. This value exceeds those reported for Zn_0_._4_Mn_0_._4_Fe_2_O_4_/PUF nanocomposite (11.1 mg g^−1^),^45^ pata-de-vaca leaves (12.7 mg g^−1^),^46^ nanosized titanate composites (8.8 mg g^−1^),^47^ amine-functionalized graphene oxide materials (10.0 mg g^−1^),^48^ and CuO-modified ceramic membrane (12.4 mg g^−1^).^51^ Although higher capacities were reported for graphene oxide-modified waste newspaper (31.3 mg g^−1^),^49^ magnetic iron-modified calcium silicate hydrate (16.3 mg g^−1^),^50^ magnetic chitosan composite (18.6 mg g^−1^),^52^ and Algerian sheep horn powder (23.8 mg g^−1^),^53^ UA-NARB offers a favorable balance between adsorption capacity, near-neutral operating pH, simple thermochemical preparation, and low-cost rice husk valorization. Its performance is therefore best appreciated not only in terms of maximum capacity, but also through the combined advantages of accessible mesoporosity, negative surface charge, oxygen-/nitrogen-containing functionalities, and practical operation under mild conditions.

**Table 4:** Comparison of Cd(ii) adsorption performance of UA-NARB with selected sorbents reported in previous studies

Sorbent type	Temp.	pH	Time, min	*q*_e_, (mg g^−1^)	Ref.
Zn_0.6_Mn_0.4_Fe_2_O_4_/PUF nanocomposite	25	6.5	60	11.1	45
Pata-de-vaca leaves	25	5.0	240	12.7	46
Nanosized titanate composites (NaxFeyMgiTiOz)	20	—	420	8.8	47
Amine-functionalized GO materials	25	5	240	10.0	48
Graphene oxide modified waste newspaper	25	6	720	31.3	49
Magnetic iron-modified calcium silicate hydrate	25	4	300	16.3	50
CuO-modified ceramic membrane	25	4	120	12.4	51
Magnetite–chitosan composite	25	5	10	18.6	52
Algerian sheep horns powder (BKASH)	25	6	30	23.8	53
UA-NARB	25	6	240	14.6	Present work

#### 3.2.4. Effect of temperature and adsorption thermodynamics

The effect of temperature was investigated to clarify the thermal response of Cd(ii) adsorption onto UA-NARB and to determine whether the uptake process is favored or inhibited by increasing temperature. The experiments were performed at 25, 30, 40, and 50 °C using 20 mg L^−1^ Cd(ii), 2.0 g L^−1^ UA-NARB, and 1.0 L solution volume. As shown in Fig. 6a, Cd(ii) removal increased slightly from 93.8% at 25 °C to 94.5% at 30 °C, then increased further to 96.6% at 40 °C and 97.4% at 50 °C. The corresponding adsorption capacity also increased mildly from 9.4 mg g^−1^ at 25 °C to 9.5, 9.7, and 9.7 mg g^−1^ at 30, 40, and 50 °C, respectively. The positive temperature effect indicates that Cd(ii) uptake by UA-NARB is slightly favored at elevated temperature. This behavior may be attributed to enhanced mobility of Cd(ii) species in solution, improved diffusion into accessible mesopores, and greater accessibility of surface interaction sites at higher temperature.^6,35^

**Fig. 6:**
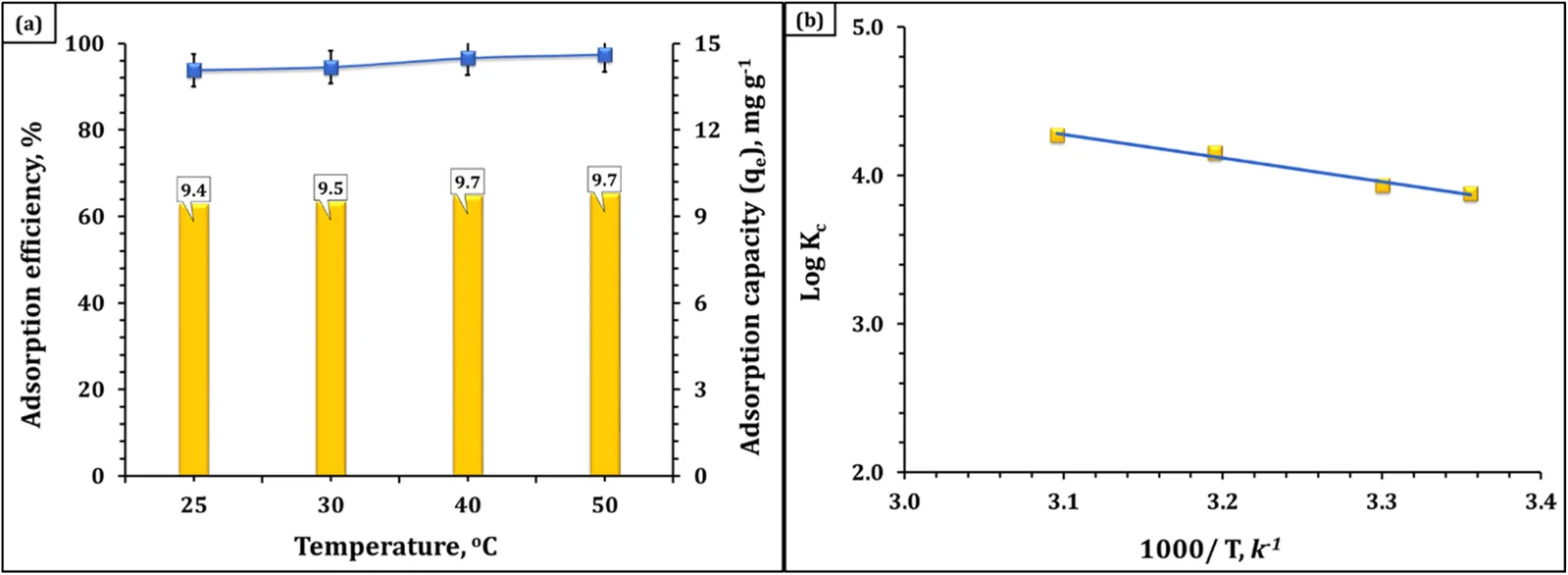
Effect of temperature and thermodynamic analysis for Cd(ii) adsorption onto UA-NARB: (a) temperature-dependent Cd(ii) removal efficiency and adsorption capacity using 20 mg L^−1^ Cd(ii) and 2.0 g L^−1^ sorbent dose; (b) Van't Hoff plot.

Thermodynamic parameters were calculated to further evaluate the spontaneity and energetic nature of Cd(ii) adsorption onto UA-.^54,55^ The thermodynamic parameters were calculated using the dimensionless equilibrium constant defined in Table S1. The Gibbs free energy change values were negative at all investigated temperatures, decreasing from −22.1 kJ mol^−1^ at 25 °C to −22.8, −24.9, and −26.4 kJ mol^−1^ at 30, 40, and 50 °C, respectively (Table S5). These negative Δ*G*° values confirm the spontaneous nature of Cd(ii) adsorption under the studied conditions. Their increasingly negative values with rising temperature further indicate that the adsorption process becomes more thermodynamically favorable at elevated temperature, in agreement with the observed improvement in removal efficiency and adsorption capacity.^38–40^

The positive enthalpy change, Δ*H*° = 30.5 ± 2.6 kJ mol^−1^, indicates that Cd(ii) adsorption onto UA-NARB is endothermic (Table S4). The van't Hoff regression showed good linearity (*R*^2^ = 0.986). This agrees with the temperature-dependent adsorption trend and suggests that higher temperature facilitates Cd(ii) transfer from the aqueous phase to the sorbent surface.^54,55^ The endothermic behavior may be associated with enhanced Cd(ii) mobility, improved diffusion within the mesoporous structure, and greater accessibility of surface interaction sites at elevated temperature.^6,35^ The positive entropy change, Δ*S*° = 176.5 ± 8.5 J mol^−1^ K^−1^, indicates increased randomness at the solid–solution interface during Cd(ii) adsorption (Table S4). This increase may arise from the release of hydration water from Cd(ii) species and surface-bound water from UA-NARB during interfacial adsorption, which can offset the ordering associated with Cd(ii) fixation on surface sites.^54,55^ Thus, the positive Δ*S*° value, together with the increasingly negative Δ*G*° values, indicates increasingly favorable thermodynamic conditions for Cd(ii) adsorption with increasing temperature.^38–40^

Overall, the temperature and thermodynamic results demonstrate that Cd(ii) adsorption onto UA-NARB is spontaneous and endothermic, with slightly improved performance at elevated temperature. The positive Δ*H*° and Δ*S*° values support a thermodynamically favorable uptake process at elevated temperature. All temperature-dependent experiments were conducted at the selected pH 6, whereas Cd^2+^ remains the predominant aqueous species; thus, Cd speciation was maintained under the selected solution-chemistry condition. It is worth noted that, the high removal achieved near ambient temperature indicates that UA-NARB can operate effectively without thermal intensification. These findings are consistent with the kinetic and isotherm results, indicating favorable occupation of finite adsorption sites supported by mesoporous diffusion and surface interactions.

### 3.3. Desorption behavior and synthetic multimetal wastewater application

Desorption experiments were conducted to evaluate the reversibility of Cd(ii) binding and to identify a suitable eluent for Cd(ii) recovery. The tests were performed using Cd(ii)-loaded UA-NARB under the preferred adsorption conditions (2.0 g L^−1^, 240 min, room temperature) with 1.0 M HCl, H_2_SO_4_, and HNO_3_ as eluents. As shown in Fig. 7a, all tested acids enabled effective Cd(ii) desorption, confirming that the adsorption process is substantially reversible under acidic conditions. The desorption efficiency followed the order H_2_SO_4_ > HNO_3_ > HCl, with values of 92.7, 81.5, and 79.1%, respectively. The high desorption efficiency, particularly with H_2_SO_4_, indicates that mineral-acid elution can effectively recover adsorbed Cd(ii) from UA-NARB. Accordingly, H_2_SO_4_ was the most effective eluent among the tested acids.

**Fig. 7:**
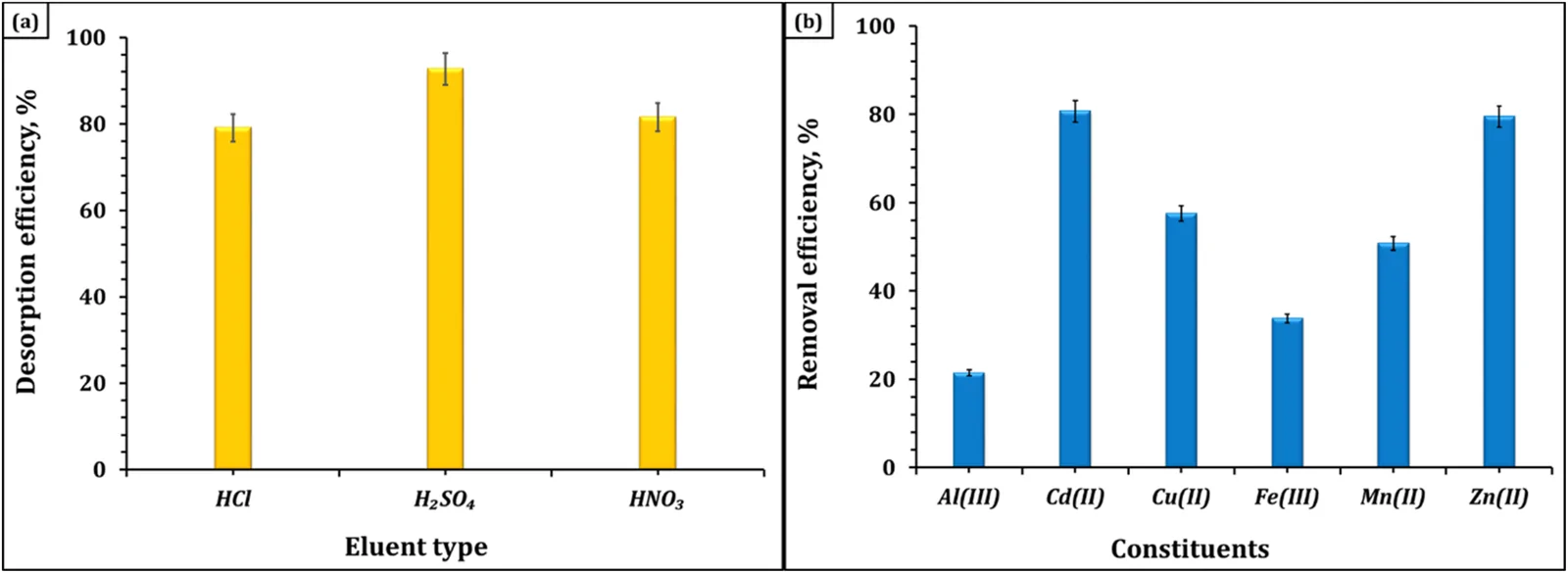
Practical performance of UA-NARB: (a) desorption efficiency using different eluents and (b) removal of coexisting metal ions from synthetic multimetal wastewater.

To evaluate performance under multicomponent conditions, UA-NARB was applied to a synthetic wastewater formulated to simulate the composition of wastewater supplied from wastewater treatment stations. The experiment was conducted under the selected treatment conditions of 2.0 g L^−1^ sorbent dose, 480 min contact time, and room temperature. The wastewater contained Al(iii), Cd(ii), Cu(ii), Fe(iii), Mn(ii), and Zn(ii) at initial concentrations of 8.0, 10.0, 10.3, 25.0, 12.0, and 11.0 mg L^−1^, respectively. After treatment with UA-NARB, the corresponding removal efficiencies were 21.4, 80.7, 57.5, 33.8, 50.8, and 79.4%, respectively (Fig. 7b). The synthetic multimetal wastewater results demonstrate that UA-NARB maintains strong Cd(ii) removal performance under competitive multicomponent conditions. Notably, Cd(ii) removal reached 80.7% in the presence of substantially higher concentrations of coexisting metal ions, demonstrating effective uptake under multicomponent conditions. The appreciable removal of Zn(ii), Cu(ii), and Mn(ii) further indicates that UA-NARB possesses broader multimetal adsorption capability, which is advantageous for practical wastewater treatment where contaminants rarely occur as isolated single species. Compared with simplified single-solute experiments, the synthetic multimetal wastewater test provides a more realistic assessment of sorbent performance. The ability of UA-NARB to retain high Cd(ii) removal in the presence of competing ions demonstrates that its adsorption sites remain accessible and effective under complex matrix conditions. This performance highlights the robustness of the sorbent and supports its suitability for treating Cd(ii)-contaminated wastewater.

Overall, the desorption and synthetic multimetal wastewater results demonstrate the practical potential of UA-NARB. Efficient Cd(ii) desorption using simple mineral acids confirms its regenerability, while strong Cd(ii) removal under multicomponent conditions highlights its applicability in simulated multicomponent wastewater matrix. These findings show that UA-NARB combines effective adsorption performance, operational practicality, and rice-husk valorization, supporting its use as a agricultural-residue-derived sorbent for Cd(ii)-contaminated wastewater treatment.

### 3.4. Proposed Cd(ii) adsorption mechanism

The adsorption of Cd(ii) onto UA-NARB can be interpreted as a cooperative process governed by pH-regulated electrostatic attraction, interaction with polar surface functionalities and mineral-associated oxygen sites, and diffusion-assisted access to internal pores, as schematically illustrated in Fig. 8. This interpretation is supported by the combined characterization and adsorption results. XRD and FTIR analyses (Fig. 1) revealed a heterogeneous carbon–mineral framework containing disordered carbon domains, polar surface functionalities, and Si–O-related surface features, while SEM and N_2_ adsorption–desorption analyses (Fig. 2) confirmed a rough mesoporous morphology that can facilitate solution transport toward adsorption sites. In addition, comparison of the FTIR spectra before and after Cd(ii) adsorption (Fig. 1b) showed measurable changes in several adsorption-relevant bands, providing direct spectroscopic evidence that the local chemical environment of surface functional groups was altered during Cd(ii) uptake. Similar multifunctional pathways have been reported for modified biochars and engineered N-containing carbonaceous sorbents used for Cd(ii) removal, where surface chemistry and porous structure operate cooperatively.^3,5,6,25,35,48,53^

**Fig. 8:**
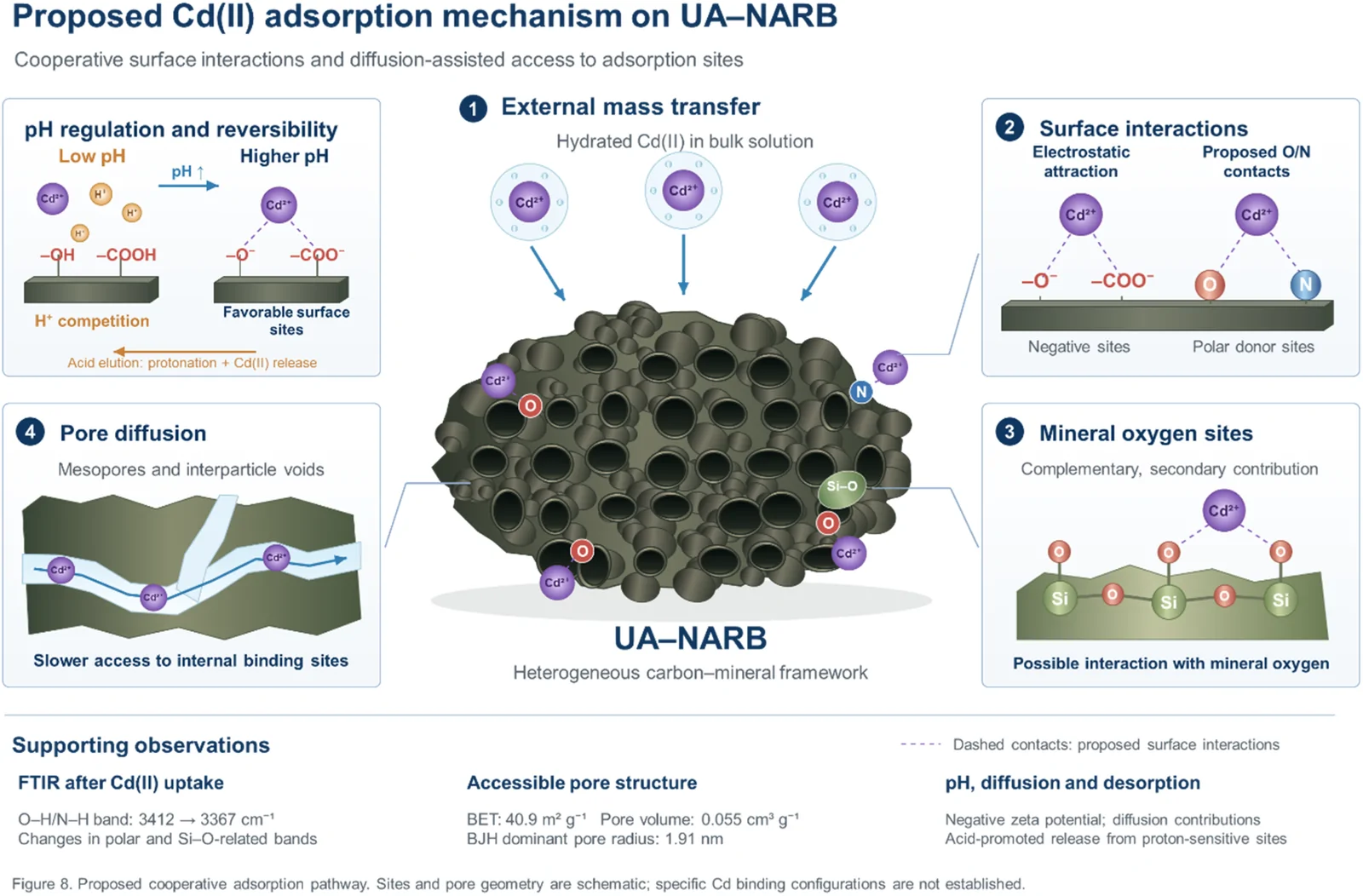
Schematic illustration of the proposed Cd(ii) adsorption pathway onto UA-NARB.

The pH-dependent adsorption behavior highlights the coupled roles of proton competition, surface charge, and functional-group availability in Cd(ii) uptake. Under strongly acidic conditions, excess H^+^ ions compete with Cd(ii) for active sites and promote protonation of hydroxyl, carboxyl/carboxylate, carbonyl-associated oxygen, C–O/C–O–C, and other polar surface groups (Fig. 1b), thereby reducing their electron-donating ability and weakening Cd(ii) interaction. With increasing pH, progressive deprotonation of these polar and mineral-associated groups generates more favorable negatively charged or electron-rich sites, enhancing Cd(ii) attraction and coordination-like surface interaction. This interpretation is consistent with the moderately negative zeta potential of UA-NARB and the FTIR evidence for O–H/N–H, carbonyl/carboxyl-type, C–O/C–N, and Si–O-containing functionalities (Fig. 1b and Table 1). The post-adsorption FTIR spectrum (Fig. 1b) further showed a shift of the broad O–H/N–H-related band from approximately 3412 to 3367 cm^−1^, together with smaller changes around 3130, 1616, 1398, 1132–1136, 1065, and 953 cm^−1^. These changes indicate that polar surface functionalities and mineral-associated domains undergo changes in their local chemical environment during Cd(ii) adsorption. Similar pH-regulated Cd(ii) adsorption involving amino, hydroxyl, carboxyl/carboxylate, and other O-/N-containing groups has been reported for modified biochars and N-doped carbonaceous adsorbents.^6,8,25,35^

The acid-desorption behavior further supports the involvement of proton-sensitive binding sites. Efficient Cd(ii) release under acidic conditions indicates that a considerable fraction of the adsorbed metal is reversibly associated with surface donor groups that can be weakened by protonation and H^+^ competition. This behavior supports adsorption through electrostatic attraction and coordination-like interaction with O-/N-containing and mineral-associated sites, consistent with the spectral changes observed after Cd(ii) loading and the high desorption efficiency achieved with mineral acids.^25,35,56^

Mineral-associated domains may provide complementary interaction sites for Cd(ii). The XRD/FTIR results indicate that UA-NARB retains inorganic oxygen-rich features, including Si–O-related domains, which can operate alongside the functionalized carbon surface. The persistence and slight modification of Si–O/mineral-associated FTIR bands after Cd(ii) uptake are consistent with involvement of these inorganic surface domains in Cd(ii) interaction. Accordingly, in UA-NARB, these domains are best considered secondary contributors alongside the polar surface functionalities.^3,5,25^

The porous structure of UA-NARB supports Cd(ii) uptake by improving mass transfer and access to internal adsorption domains. UA-NARB displayed a BET surface area of 40.9 m^2^ g^−1^, a total pore volume of 0.055 cm^3^ g^−1^, and a BJH dominant pore radius of 1.91 nm (Table 1), confirming a moderately developed mesoporous structure. Although the surface area is not extremely high, mesopores and interparticle voids can facilitate the movement of hydrated Cd(ii) species toward internal binding sites. This textural contribution agrees with the contact-time profile, where rapid uptake occurred during the initial stage followed by slower adsorption at longer contact time. It is also consistent with the Weber–Morris and Bangham analyses, which indicate contributions from intraparticle and pore diffusion during Cd(ii) uptake (Fig. 4). Similar diffusion-assisted Cd(ii) uptake has been reported for N-doped porous carbon and functionalized biochar systems, where pore structure facilitates transport while surface sites govern binding strength.^6,7^

The kinetic and equilibrium modeling results provide complementary descriptions of the adsorption behavior. The better agreement of the pseudo-second-order model indicates that the adsorption rate is strongly associated with the availability of active sites on UA-NARB. When considered together with the diffusion-model results, the kinetic behavior is consistent with rapid external binding followed by slower diffusion into internal mesoporous domains. The Langmuir and Sips models describe finite adsorption capacity and heterogeneous surface behavior, respectively, consistent with the structurally heterogeneous carbon–mineral framework identified by XRD, FTIR, and surface-texture analyses. These findings agree with the heterogeneous carbon–mineral structure revealed by characterization and indicate that Cd(ii) adsorption occurs on a limited number of accessible but non-identical binding sites.^6,8,35^ Thermodynamic results further describe the energetic behavior of the adsorption process. The negative Δ*G*° values confirm spontaneous Cd(ii) adsorption, while the positive Δ*H*° value indicates an endothermic process. Similar spontaneous and endothermic Cd(ii) adsorption has been reported for amino-modified biochar and N-doped hierarchical porous carbon.^6,35^ The positive Δ*S*° value suggests increased randomness at the solid–solution interface during adsorption. Overall, the thermodynamic behavior is consistent with favorable Cd(ii) uptake accompanied by interfacial interactions with the functionalized biochar surface.

Based on these combined findings and the proposed schematic representation in Fig. 8, Cd(ii) adsorption onto UA-NARB can be summarized as a four-step cooperative process. First, Cd(ii) ions are transported from the bulk solution to the external surface of UA-NARB by concentration-gradient-driven mass transfer. Second, Cd(ii) interacts with negatively charged and polar surface sites through electrostatic attraction and coordination-like surface interactions. Third, mineral-associated oxygen sites, including Si–O-related domains inherited from rice husk, may provide additional interaction centers. Fourth, mesopores and interparticle voids facilitate diffusion toward less accessible internal sites. The post-adsorption FTIR changes, pH-dependent uptake, negative surface charge, diffusion behavior, and acid-desorption response collectively support this cooperative interaction pathway. Overall, the combined evidence is consistent with a cooperative adsorption process involving surface chemistry, pH-regulated charge interactions, mesoporous accessibility, and diffusion-assisted transport.

## 4. Conclusion

This study demonstrated the successful preparation of urea/acrylamide-assisted nitrogen-functionalized activated rice husk biochar, UA-NARB, as a agricultural-residue-derived sorbent for Cd(ii) removal from aqueous systems. The applied K_2_CO_3_-assisted activation and urea/acrylamide-assisted nitrogen-functionalization route yielded a heterogeneous carbon–mineral sorbent containing disordered carbon domains, oxygen-/nitrogen-containing surface functionalities, mineral-associated oxygen sites, accessible mesoporosity, and a moderately negative surface charge. These structural and surface properties provided a favorable platform for Cd(ii) uptake through cooperative surface interactions and diffusion-assisted transport. Adsorption performance was strongly governed by solution chemistry, sorbent loading, contact time, and initial Cd(ii) concentration. Cd(ii) removal increased markedly with pH, and pH 6 was selected as the working condition because it provided high removal under near-neutral conditions while avoiding unnecessary alkalinization. The dose study showed that 2.0 g L^−1^ offered the best balance between removal efficiency and sorbent utilization. Kinetic analysis revealed rapid initial Cd(ii) capture followed by gradual equilibration, with the pseudo-second-order model providing the best empirical description and diffusion models confirming the contribution of intraparticle and pore diffusion. Equilibrium adsorption was best represented by the Langmuir and Sips models, giving maximum capacities of 14.6 and 15.1 mg g^−1^, respectively, and confirming favorable finite-site adsorption on a moderately heterogeneous surface. Thermodynamic analysis indicated that Cd(ii) uptake was spontaneous and endothermic, with adsorption becoming more favorable at elevated temperature. Beyond synthetic solutions, UA-NARB showed promising practical performance. Efficient Cd(ii) desorption was achieved using simple mineral acids, particularly 1.0 M H_2_SO_4_, which released 92.7% of the adsorbed Cd(ii). In synthetic multimetal wastewater, UA-NARB maintained strong Cd(ii) removal of 80.7% despite the presence of competing metal ions, highlighting its applicability under realistic aqueous conditions. The proposed mechanism involves pH-regulated electrostatic attraction, interaction with hydroxyl, carboxyl/carboxylate, carbonyl, C–O/C–O–C, nitrogen-containing, and Si–O-related sites, contribution from mineral-associated oxygen domains, and diffusion-assisted access through mesopores and interparticle voids. Although rice husk provides a renewable waste-derived precursor, the overall environmental performance of the preparation route also depends on chemical consumption, thermal energy demand, washing requirements, and the use of acrylamide as a nitrogen precursor; these factors should be considered in future process-scale evaluation. Overall, UA-NARB demonstrates effective Cd(ii) adsorption together with the valorization of rice husk as an agricultural residue, supporting its potential for further development in wastewater-treatment applications.

## Author contributions

Adel El-Zahhar: material synthesis, validation, data interpretation, writing & editing the original draft. Majed Alghamdi: material synthesis validation, data curation, reviewing the original draft.

## Conflicts of interest

The authors declare that they have no known financial or non-financial interests, direct or indirect, related to the submitted work within the past three years or beyond, that could reasonably be perceived as influencing the manuscript.

## Supplementary Material

RA-016-D6RA08271H-s001

## Data Availability

The datasets used and/or analyzed during the current study are available from the corresponding author on reasonable request. Supplementary information (SI): additional experimental details, characterization data, figures, and tables supporting the findings of this study. See DOI: https://doi.org/10.1039/d6ra08271h.
