# Supplementary material for: Urea/acrylamide-assisted nitrogen-functionalized rice husk biochar for Cd(ii) removal: adsorption performance and interaction insights
Source: RSC Adv. 2026 Oct 8;16(46):60886–902. doi: 10.1039/d6ra08271h (PMC13646914; doi:10.1039/d6ra08271h)
Supplement: Supplementary file 1 [file RA-016-D6RA08271H-s001.pdf]

Supplementary Information (SI) for RSC Advances.
This journal is © The Royal Society of Chemistry

Urea/Acrylamide-Assisted Nitrogen-Functionalized Rice Husk Biochar for Cd(II) Removal: Adsorption Performance and Interaction Insights

Adel A. El-Zahhar[1], Majed M. Alghamdi[1]*

[1] *Department of Chemistry, Faculty of Science, King Khalid University, P.O.Box 9004, Abha 61413, Saudi Arabia*

* Corresponding author: Azmasnew26@hotmail.com

Section S1. Batch adsorption and analytical quality control

Each adsorption condition was prepared independently in triplicate, and the results are reported as mean ± standard deviation (SD). Error bars represent ±SD, and the relative standard deviation (RSD) of the replicate measurements did not exceed 5%.

Cd(II) concentrations were determined using a GBC 932 AA atomic absorption spectrophotometer at 228.8 nm. Calibration was performed using a reagent blank and five non-zero Cd(II) standards covering the range 0.2–1.6 mg L⁻^{1}, each measured in triplicate. Linear regression was performed using the mean responses of the non-zero standards, while the blank was not forced through the regression. The resulting calibration equation was A = 0.08524C + 0.00220, with R^2 = 0.9984. The limits of detection and quantification, calculated from the residual standard deviation of the calibration regression using LOD=3.3σ/S and LOQ=10σ/S, were 0.09 and 0.26 mg L⁻^{1}, respectively. Procedural blanks and mid-range quality-control standards were analyzed with each analytical batch, and the quality-control recoveries remained within 95–105%. Samples exceeding the calibration range were appropriately diluted before analysis.

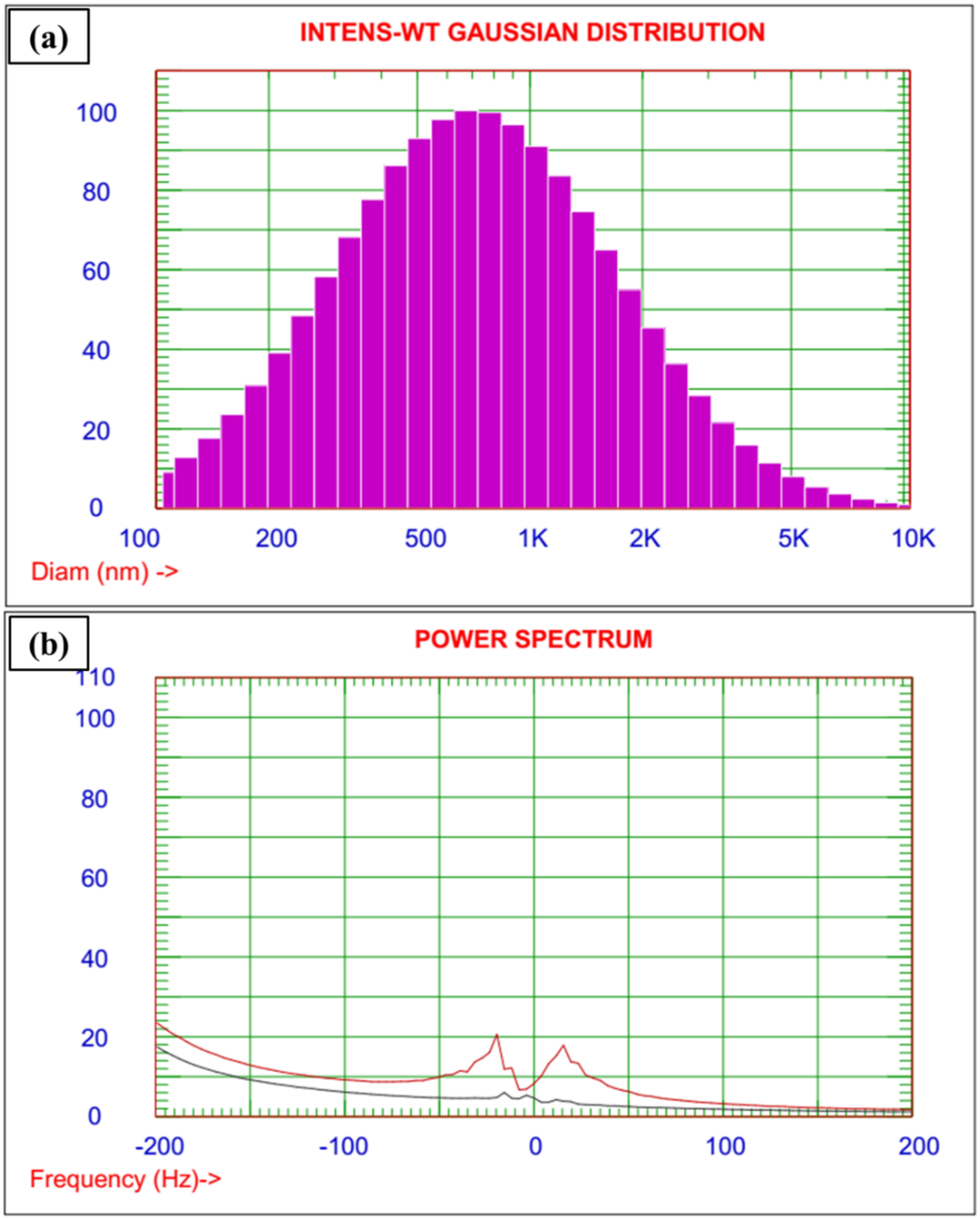

Figure S1. *DLS analysis of UA–NARB dispersed in water: (a) intensity-weighted hydrodynamic particle-size distribution and (b) corresponding power spectrum.*

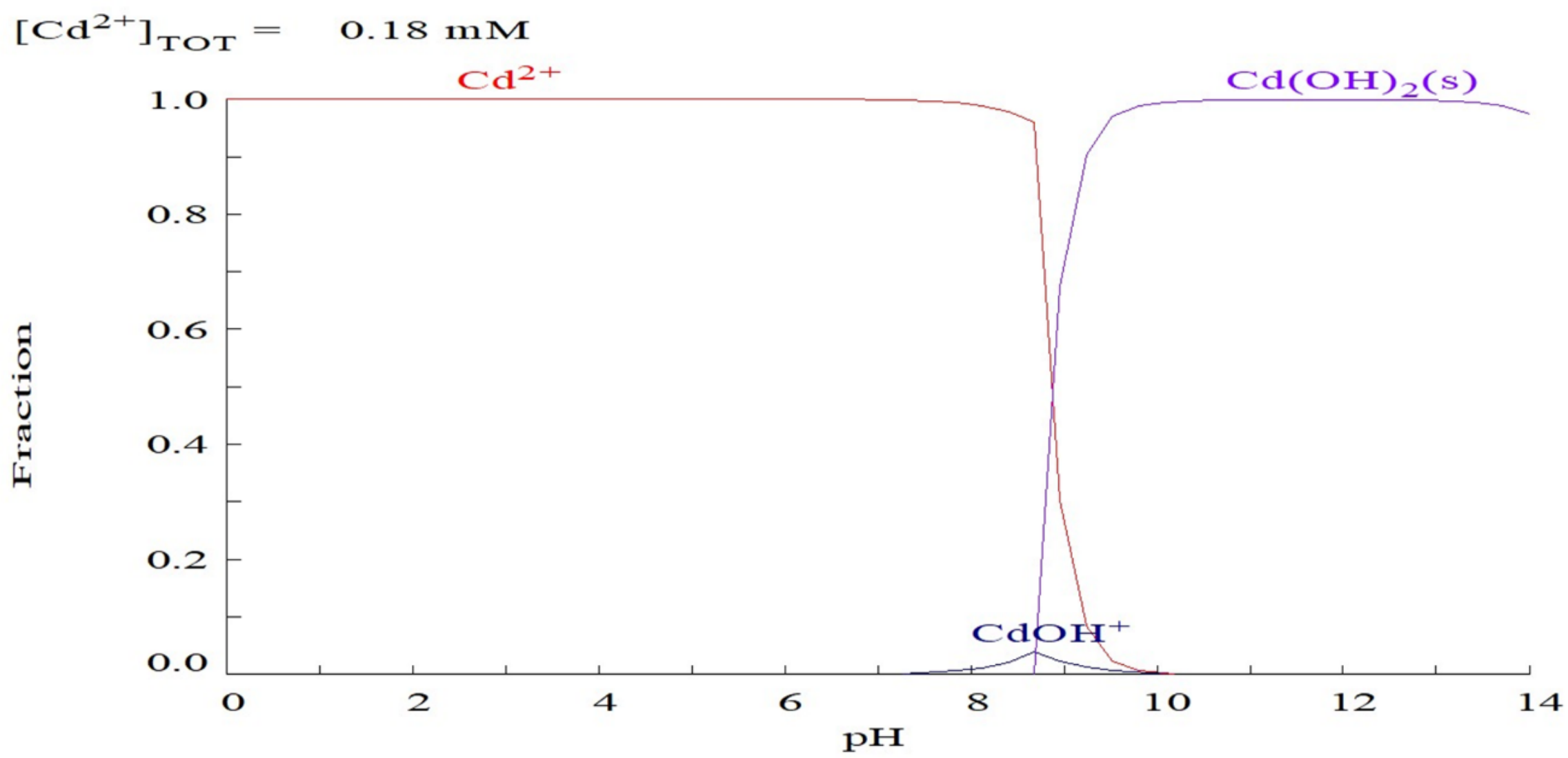

Figure S2: *Expected aqueous speciation of Cd(II) at 20 mg L*⁻*^{1} (0.178 mM) as a function of pH, calculated using MEDUSA/HYDRA.*

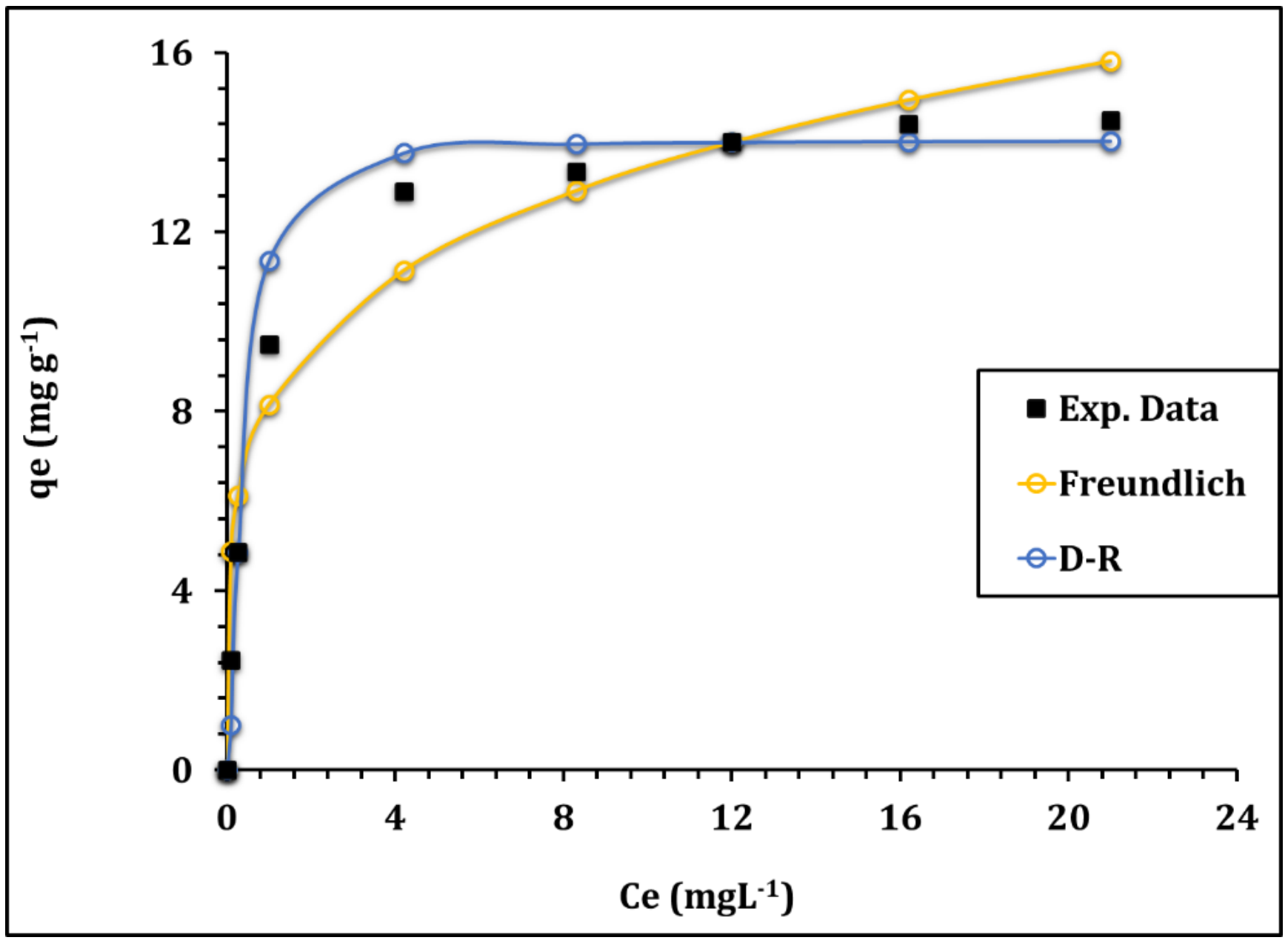

Figure S3. *Freundlich and Dubinin–Radushkevich isotherm fitting for Cd(II) adsorption onto UA–NARB.*

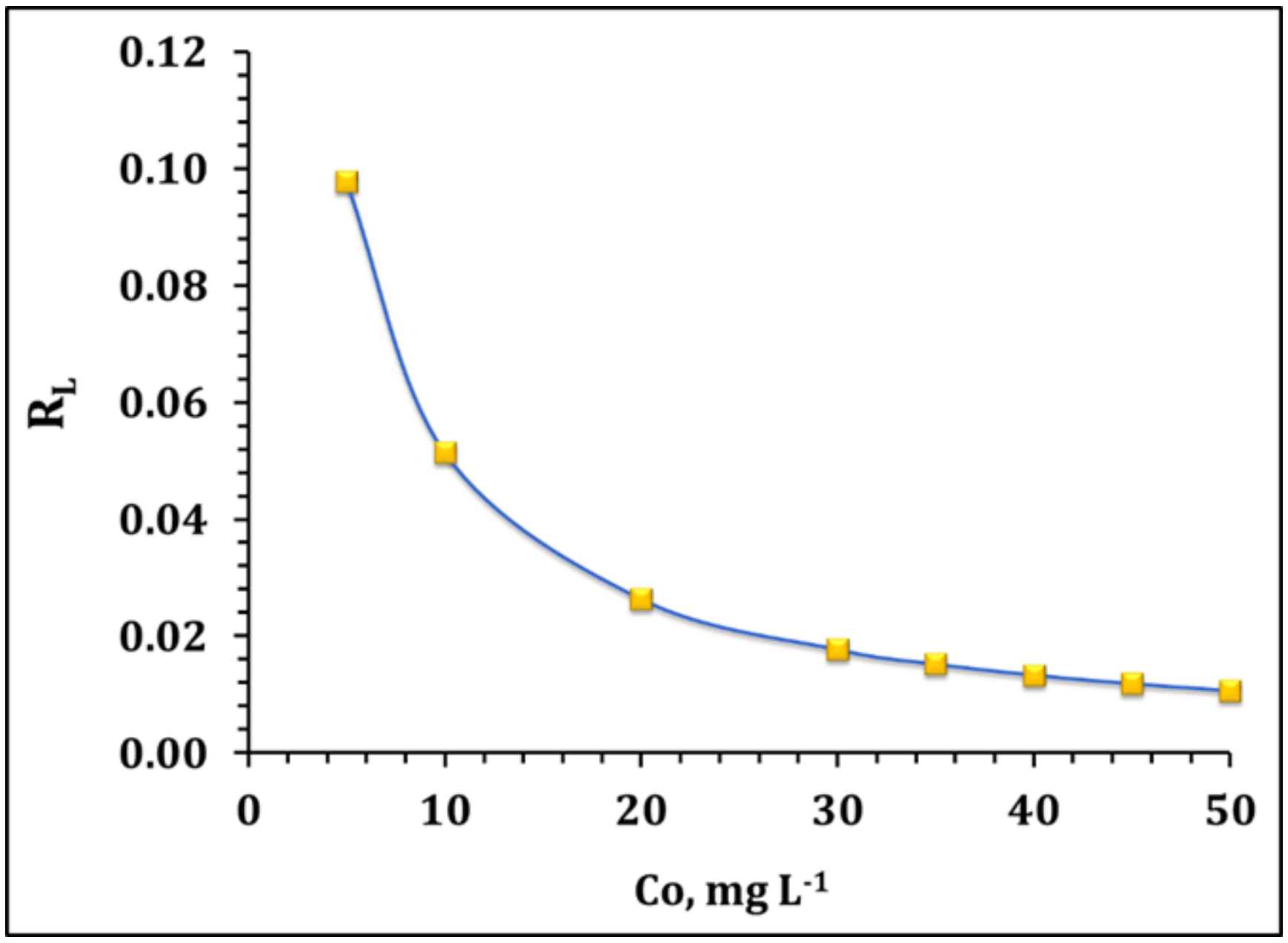

Figure S4. *Variation of the Langmuir separation factor (R_L) with initial Cd(II) concentration for adsorption onto UA–NARB.*

Table S1: *Kinetic, isotherm, thermodynamic, and fitting equations used for Cd(II) adsorption onto UA–NARB [1-7].*

Kinetics	Equations
**Pseudo-first-order**	$q_t = q_1(1 - e^{-k_1 t})$
**Pseudo-second-order**	$q_t = \frac{k_2 q_e^2 t}{1 + k_2 q_e t}$
**Intra-particle diffusion model (IPD)**	$q_t = K_{id}\, t^{0.5} + C_i$
**Bangham**	$\log_{10}\left(\log_{10}\left(\frac{C_o}{C_o - q \times m}\right)\right) = \log_{10}\left(\frac{k_o m}{2.303\, V}\right) + \square \log_{10} t$
**Isotherms**	**Equations**
**Langmuir model**	$q_e = \frac{q_m k_L C_e}{1 + k_L C_e}$
**Freundlich model**	$q_e = K_F C_e^{1/n_F}$
**Dubinin-Radushkevich (D_R) model**	$\ln q_e = \ln q_S + K_{ad}\square^2$
**Sips model**	$q_e = \frac{q_S (K_S C_e)^{mS}}{1 + (K_S C_e)^{mS}}$
**Thermodynamics**	**Equations**
	$\log K_C = -\frac{\square H^o}{2.303\, R} \times \frac{1}{T} + C$ $-\square G^o = 2.303\, RT \log K_C$ $\square G^o = \square H^o - T \square S^o$
**Fitting**	**Equations**
**Coefficient of determination** (R^2)	$R^2 = 1 - \frac{\square_1^n (q_{exp} - q_{pred})^2}{\square_1^n \left(q_{exp} - \overline{q_{exp}}\right)^2}$
**Chi-square error function** ($\square^2$)	$X^2 = \square_{i=1}^{n} \frac{(q_{exp,i} - q_{cal,i})^2}{q_{cal,i}}$

qe (mg g⁻¹) is the equilibrium adsorption capacity, qt (mg g⁻¹) is the adsorption capacity at time t (min), and Ce (mg L⁻¹) is the equilibrium concentration of Cd(II) in solution. k_1 (min⁻¹) and k_2 (g mg⁻¹ min⁻¹) are the rate constants for the pseudo first and second order, respectively. k_{id} (mg g⁻¹ min⁻¹/²) is a rate constant, and C is the thickness of the boundary layer, K_o and □ are constants. qₘ and q_S (mg g⁻¹) are the Langmuir and Sips saturation capacities; K_L (L mg⁻¹) is the Langmuir constant; R_L is dimensionless; K_F has units mg g⁻¹ (L mg⁻¹)(1/n_F); n_F is the Freundlich intensity exponent. K_{ad} (mol²/J²) is the D-R constants; The mean sorption energy (E_{DR}) which equals $(1/\sqrt{2K_{ad}})$; □ is Dubinin–Radushkevich isotherm constant and equal $(RT \ln(1 + 1/C_e)$ **[5-7]**. K_S [(L mg⁻¹)(m_S)] and m_S are the Sips constant and exponent. *KC is the dimensionless thermodynamic equilibrium constant and was calculated from K_d × ρ × 1000* ***[4-5]****; Here, Kd = qe/Ce (L g□¹), so multiplication by ρ and 1000 yields a dimensionless equilibrium constant. T is the temperature (K), □ is the universal gas constant (8.314 J mol⁻¹. K⁻¹), ρ is the solution density (approximately 1.0 g mL□¹), and the factor 1000 converts mL to L. R² and □² are the coefficient of determination and chi-square error function, respectively; n is the number of experimental points, qe,exp is the experimental equilibrium adsorption capacity, and qe,cal is the model-predicted adsorption capacity.*

References:

1. M. E. Gonzalez-Lopez, C. M. Laureano-Anzaldo, A. A. Pérez-Fonseca, M. Arellano and J. R. Robledo-Ortiz, *Sep. Purif. Rev.*, 2022, **51**, 358–372.
2. M. Musah, Y. Azeh, J. T. Mathew, M. T. Umar, Z. Abdulhamid and A. I. Muhammad, *CaJoST*, 2022, **4**, 20–26.
3. M. H. Taha, *Environ. Sci. Pollut. Res.*, 2021, **28**, 12475–12489.
4. Y. Wang, C. Wang, X. Huang, Q. Zhang, T. Wang and X. Guo, *Chemosphere*, 2024, **349**, 140736.
5. Q. Hu, R. Lan, L. He, H. Liu and X. Pei, *J. Environ. Manage.*, 2023, **329**, 117104.
6. A. M. Masoud, H. Ammar, A. A. Elzoghby, H. H. El Agamy and M. H. Taha, *J. Rare Earths*, 2025, **43**, 1502–1512.
7. C. R. Girish, *Chem. Pap.*, 2025, **79**, 5687–5706.

Table S2. *Initial and final pH values recorded during the Cd(II) adsorption experiments with UA–NARB.*

Initial pH	Final pH
2.03	2.05
3.01	3.00
4.00	4.05
4.99	4.94
6.01	6.02
7.98	8.01

Table S3. *Weber–Morris intraparticle diffusion and Bangham model parameters for Cd(II) adsorption onto UA–NARB.*

Weber and Morris model	**Stage I**	
	k_i (mg/g min$^{1/2}$)	0.98
	C	0.10
	R^2	0.98
	**Stage II**	
	k_i (mg/g min$^{1/2}$)	0.13
	C	6.60
	R^2	0.97
Bangham's equation model	k_o (mg/g min)	0.06
	□	0.52
	R^2	0.96

Table S4. *Thermodynamic parameters for Cd(II) adsorption onto UA–NARB.*

□G° (kJ mol^{-1})				□H°	□S°
**25 °C**	**30 °C**	**40 °C**	**50 °C**	**(kJ mol^{-1})**	**(J mol^{-1} K^{-1})**
-22.1	-22.8	-24.9	-26.4	30.5 ± 2.6	176.5 ± 8.5

Van't Hoff regression: R^2 = 0.986.
